# Supplementary material for: Mechanically interlocked networks cross-linked by a molecular necklace
Source: Nat Commun. 2022 Mar 16;13:1393. doi: 10.1038/s41467-022-29141-7 (PMC8927564; doi:10.1038/s41467-022-29141-7)
Supplement: Supplementary file 1 — Supplementary Information [file 41467_2022_29141_MOESM1_ESM.pdf]

Supporting Information for

Mechanically interlocked networks cross-linked by a molecular necklace

Zhaoming Zhang[1], Jun Zhao[1], Zhewen Guo[1], Hao Zhang[1], Hui Pan[1], Qian Wu[1], Wei You[1], Wei Yu[1], and Xuzhou Yan[1*]

[1]School of Chemistry and Chemical Engineering, Frontiers Science Center for Transformative Molecules, Shanghai Jiao Tong University, Shanghai 200240, P. R. China

[*]To whom correspondence should be addressed. E-mail: xzyan@sjtu.edu.cn

Materials and general methods

All reagents were commercially available and used as supplied without further purification. Deuterated solvents were purchased from Cambridge Isotope Laboratory (Andover, MA). Compounds **2**[1], **3**–**7**[2], **9**[3], **10**[4], **11**–**12**[5], and metallacycle[6] were prepared according to the established methods. For these reported compounds, only ^{1}H NMR spectra were measured and compared with those in literatures to confirm their structures. NMR spectra were recorded with a Bruker Avance DMX 400 spectrophotometer with use of the deuterated solvent as the lock and the residual solvent or TMS as the internal reference. Melting point of solid compounds were tested on a WRX-4 micro melting point apparatus (Shanghai Yice instrument Co., LTD, China). High resolution mass spectra were obtained on a Bruker SolariX 7.0T FT-ICR MS spectrometer. Gel permeation chromatograph (GPC) was measured by a HLC-8320GPC (TOSOH, Japan) instrument using dimethylformamide (DMF) as eluent with polystyrene standards. Scanning electron microscopy (SEM) and energy-dispersive X-ray (EDX) mapping were collected by a JSM-7800F field emission scanning electron microscope. Synchrotron radiation small angle X-ray scattering (SAXS) patterns were obtained at BL16B1 beamline, SSRF (energy ring 3.5 GeV, beam current 220 mA). The FTIR spectroscopy was recorded with Nicolet 6700 FT-IR spectrophotometer. The thermal stability analysis was conducted using a TA Instruments Q500 thermogravimetric analyzer (TGA) under the nitrogen. Each sample (~5 mg) was heated from 50 to 800 °C with a rate of 20 °C/min. Transition temperatures of materials were determined on a TA Instruments Q2000 differential scanning calorimetry (DSC) under the nitrogen. Dynamic thermomechanical analysis (DMA) was carried out with a dynamic mechanical thermal analyzer (Discovery DMA 850, TA, USA) in the tensile mode at a frequency of 1.0 Hz. Rheological experiments were carried out using a Kinexus Ultra rotational rheometer (NETZSCH Instruments) and a TA Instruments ARES G2 stress-controlled rheometer with an 8.0 mm parallel plate attachment.

Mechanical tests: The mechanical properties of the polymers were measured using an Instron 3343 machine in standard stress/strain experiments. Young's modulus was determined from the initial slope of the stress–strain curves. Toughness was obtained by integrating the area under the stress–strain curve.

Fitting of SAXS data: The collected SAXS data were reduced and analyzed using the SASfit software package.[7] The best fit for the scattering curve was a sum of two models, the power law model and the Beaucage Exponential Power Law model.[8,9] The power law model is primarily used to show the presence of a larger entangled network, and describes the scattering intensity as $I(q) = Aq^{-n}$. The Beaucage model refers to a system with multiple-size-scale feature, which is suitable for describing the metallacycle surrounded by crown ethers. The large-scale structure of size R_g corresponds to the metallacage while the small size R_s corresponds to the crown ether. The functions are given by:

$$I(q) = Aq^{-n} + Gexp\left(-\frac{Q^2R_g^2}{3}\right) + B\exp\left(-\frac{Q^2R_s^2}{3}\right)\left(\frac{[\mathrm{erf}\,(QkR_g/\sqrt{6})]^3}{Q}\right)^P$$

$$+ G_s exp\left(-\frac{Q^2R_s^2}{3}\right) + B_s\left(\frac{[\mathrm{erf}\,(Qk_sR_s/\sqrt{6})]^3}{Q}\right)^{P_s} + C$$

Constrained geometries simulate external force (CoGEF) calculations: The CoGEF simulations were performed on Spartan'14 following Beyer's method.[10] To clarify the force induced dynamicity process, the molecular necklace pattern of MIN-**2** was simplified as one DB24C8 subring threaded on an arm of metallacycle. Analogously, the control-**2** was simplified as the subring threaded on half of the cross-linker. Each structure was built based on the crystal structure[11] and minimized using molecular mechanics (MMFF). The distance between the terminal methyl groups was constrained and increased by increments of 0.2 Å. At each step, the energy was minimized by molecular mechanics (MMFF) via Spartan'14 in vacuum without counterions, and then DFT via Gaussian09 (G09)[12] with the Becke three-parameter hybrid exchange and the Lee−Yang−Parr correlation functional (B3LYP).[13,14] The 6-31G* basis set[15] was used for H, C, N, O and P atoms, while the Los Alamos National Laboratories (LANL2DZ) basis set[16] and pseudopotential was used for Pt. The relative energy of each intermediate was determined by setting the energy of the initial state at 0 kcal/mol.

Synthetic routes of the compounds used in this study

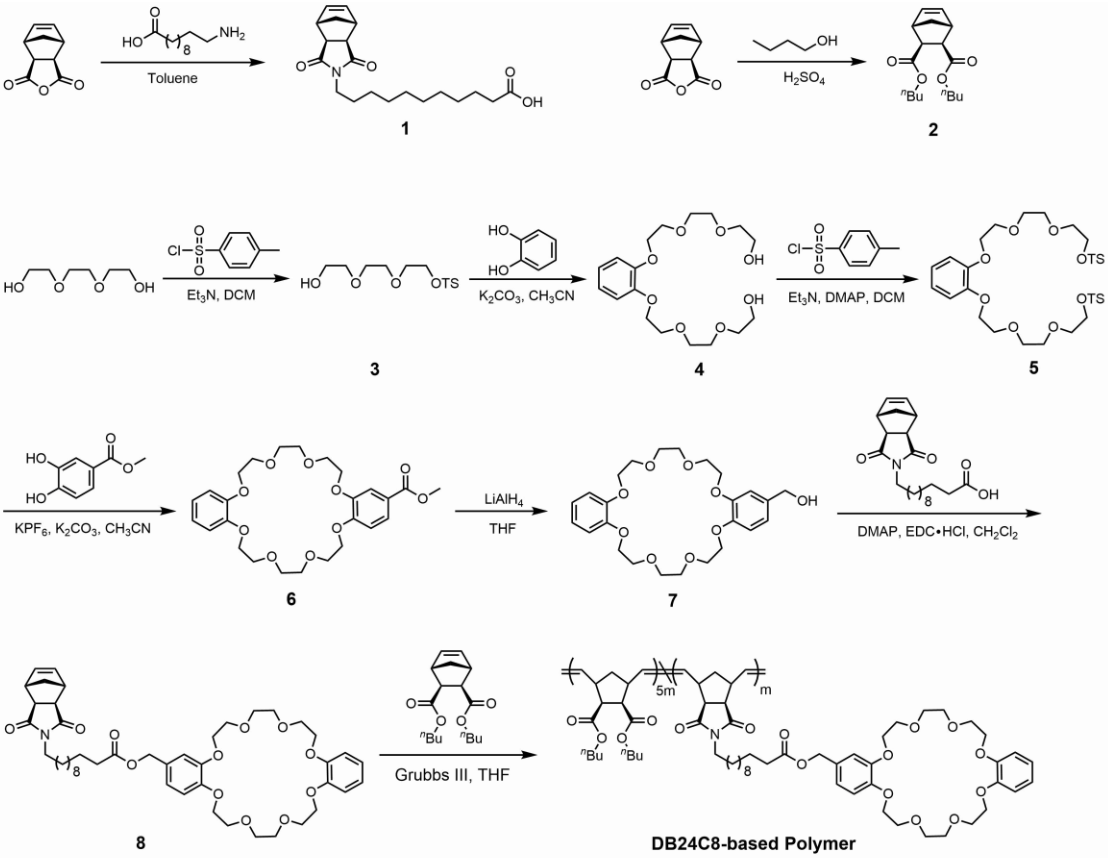

Supplementary Fig. 1 Synthetic route of the DB24C8-based polymer.

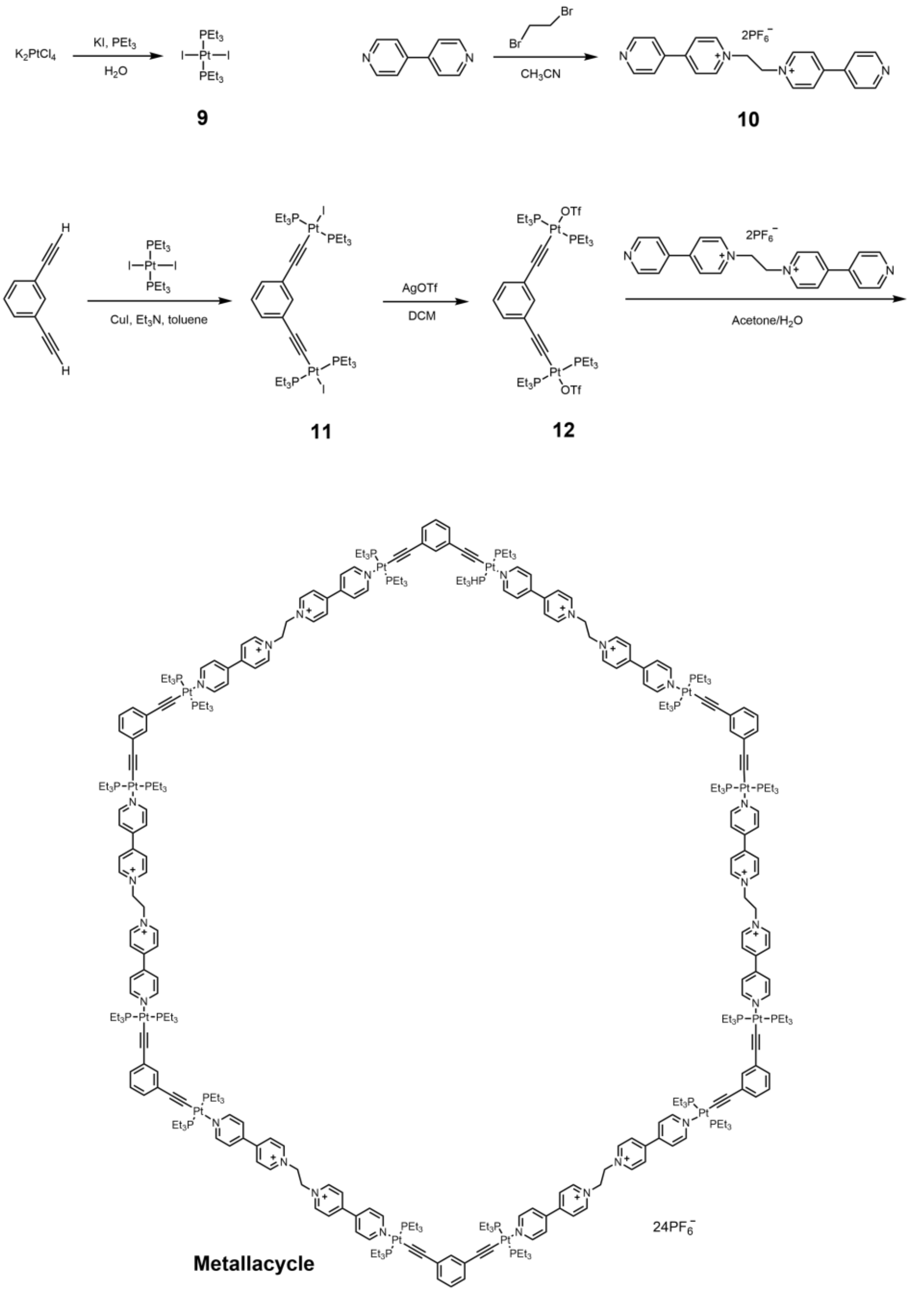

Supplementary Fig. 2 Synthetic route of the coordination-driven metallacycle.

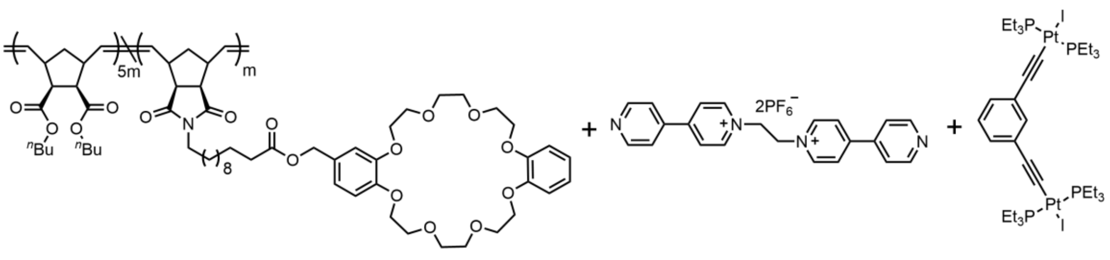

Supplementary Fig. 3 Constitution of the control-1 sample. In this case, platinum coordinated with iodine cannot interact with the bis(pyridinium) ligand. Hence, no significant cross-links exist in the control sample.

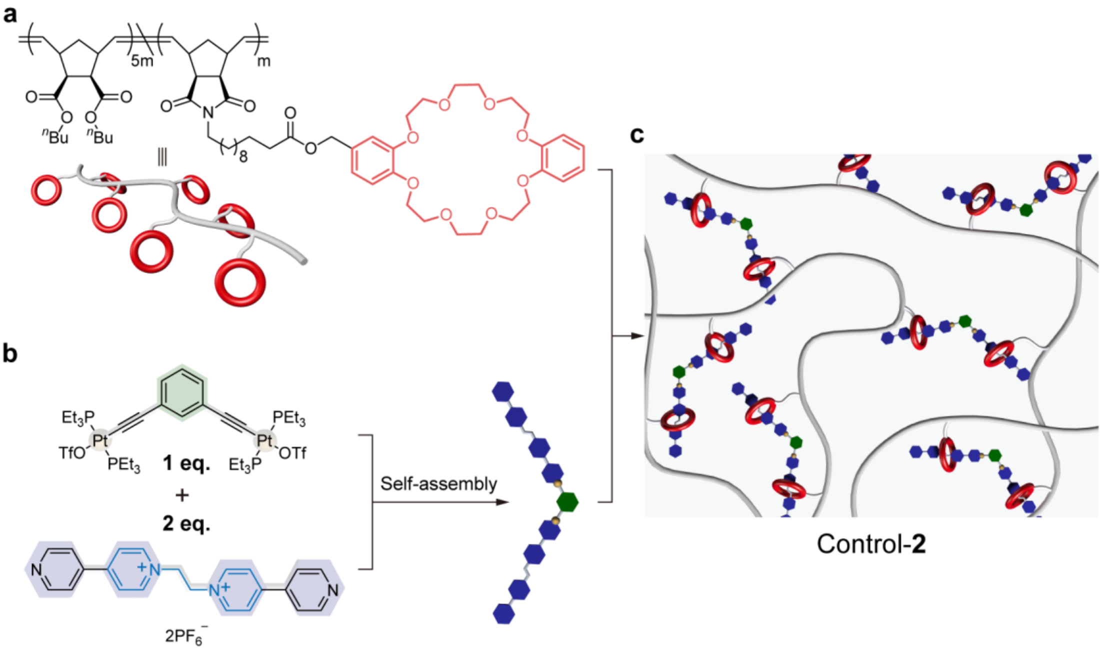

Supplementary Fig. 4 Structure of the control-2 sample. Chemical structures and corresponding cartoons of DB24C8-based polymer (**a**) and the assembly (**b**). **c** Schematic representation of control-**2** cross-linked by [2]pseudorotaxane moieties. The complex composed of a diplatinum acceptor coordinated with two bis(pyridinium) ligand could crosslink the polymer through the formation of two [2]pseudorotaxane moieties, and such control-**2** can be regarded as a general supramolecular polymer network.

Synthesis of compound 1

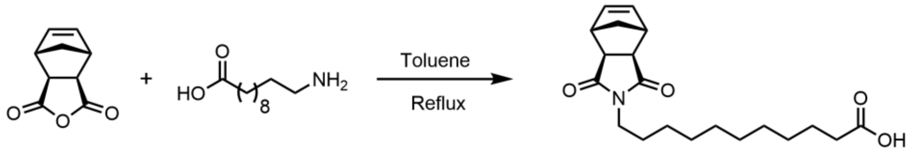

1

cis-5-Norbornene-*exo*-2,3-dicarboxylic anhydride (3.28 g, 20.0 mmol) and 11-aminoundecanoic acid (4.83 g, 24.0 mmol) in toluene (100 mL) were added to a round-bottom flask equipped with a Dean–Stark trap and a magnetic stirrer. The mixture was reacted under a nitrogen atmosphere at 120 °C for 24 h. Once the reaction was complete, it was cooled and then concentrated under reduced pressure. The residue was purified by column chromatography on silica gel with hexane/ethyl acetate = (1/1 v/v) as an eluent to afford compound **1** as a white solid (5.70 g, 82%). M.p. = 55 °C. The ^{1}H NMR spectrum of compound **1** is shown in Supplementary Fig. 5. ^{1}H NMR ($CDCl_3$, room temperature, 400 MHz) 10.48 (s, 1H), 6.26 (t, *J* = 2.0 Hz, 2H), 3.51–3.35 (m, 2H), 3.25 (t, *J* = 1.8 Hz, 2H), 2.65 (d, *J* = 1.2 Hz, 2H), 2.32 (t, *J* = 7.6 Hz, 2H), 1.65–1.55 (m, 2H), 1.55–1.45 (m, 2H), 1.35–1.16 (m, 14H). The ^{13}C NMR spectrum of compound **1** is shown in Supplementary Fig. 6. ^{13}C NMR ($CDCl_3$, room temperature, 100 MHz) δ (ppm): 179.76, 178.19, 137.81, 47.79, 45.15, 42.70, 38.74, 34.05, 29.32, 29.26, 29.14, 29.06, 29.00, 27.73, 26.90, 24.66. HRESIMS is shown in Supplementary Fig. 7: *m*/*z* calcd for $C_{20}H_{29}NO_4$, 348.2196 $[M + H]^+$; found 348.2180 $[M + H]^+$.

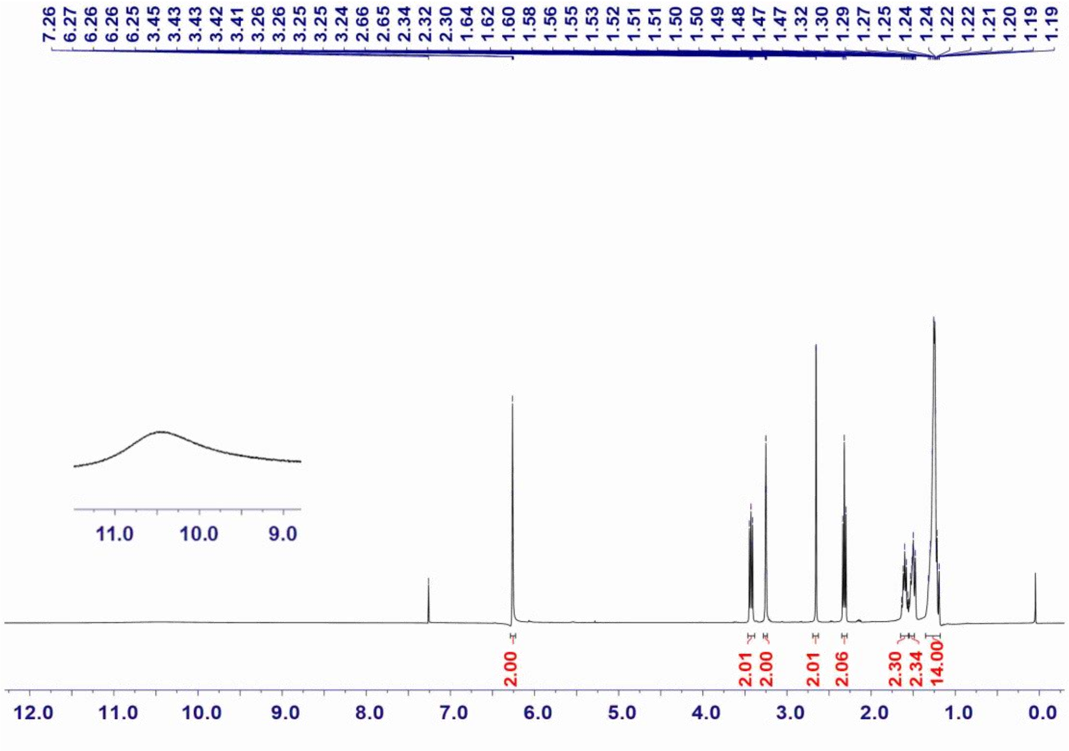

Supplementary Fig. 5 ^{1}H NMR spectrum ($CDCl_3$, room temperature, 400 MHz) of compound **1**.

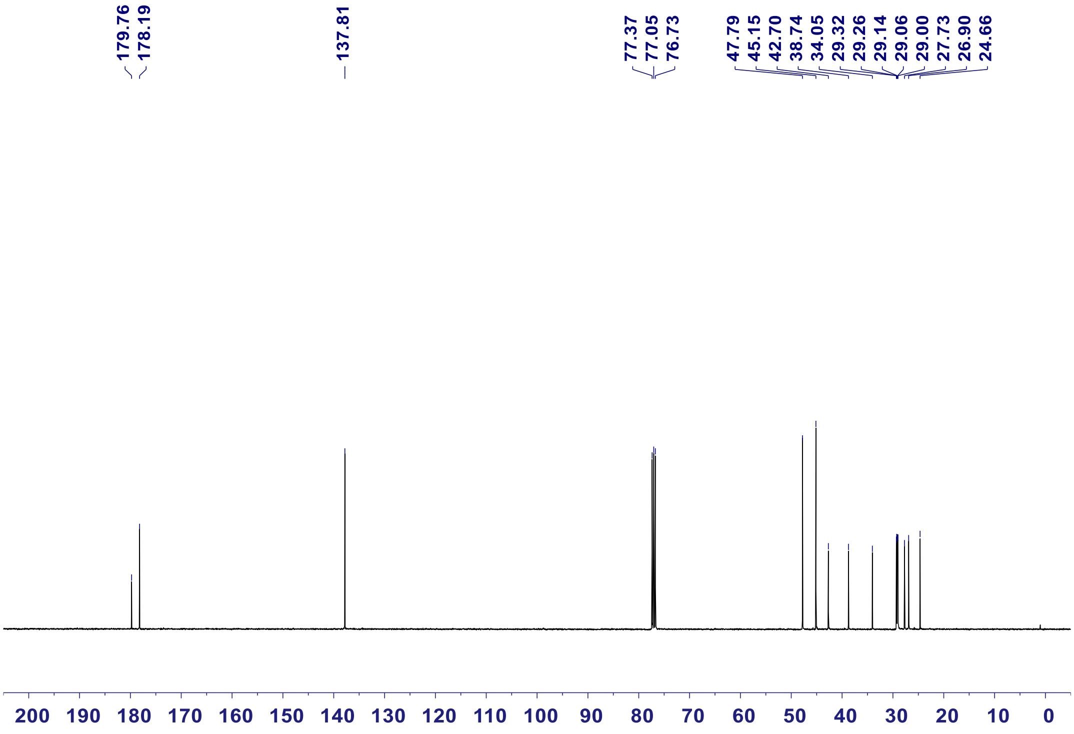

Supplementary Fig. 6 ^{13}C NMR spectrum ($CDCl_3$, room temperature, 100 MHz) of compound **1**.

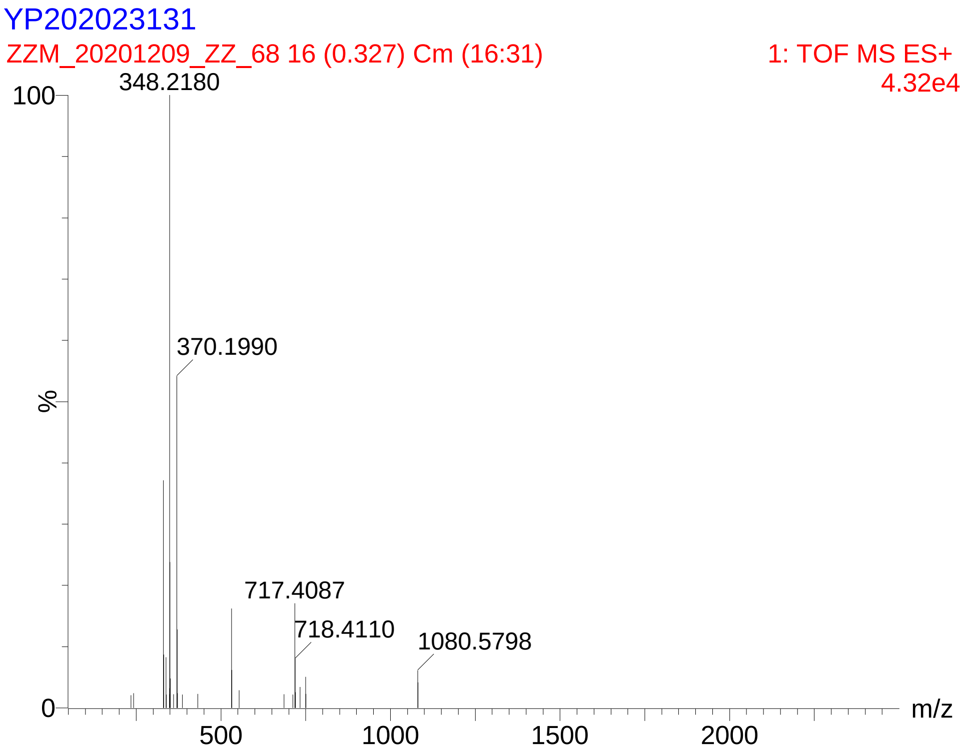

Supplementary Fig. 7 Electrospray ionization mass spectrum of compound **1**.

Synthesis of compound 2 (ref. 1)

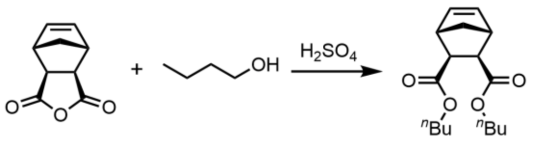

2

cis-5-Norbornene-*exo*-2,3-dicarboxylic anhydride (2.00 g, 12.2 mmol), 4 drops of concentrated sulfuric acid, and 20 mL of the corresponding anhydrous *n*-butyl alcohol was stirred under air at 75 °C. After 20 h, the colorless solution was cooled to room temperature and was concentrated under reduced pressure. The resulting pale-yellow oil was re-dissolved in 50 mL of CH_2Cl_2 and washed with saturated aqueous $NaHCO_3$ and brine. The organic solution was dried over $MgSO_4$, filtered, and concentrated in vacuo. The crude was further purified via gel chromatography with hexane as an eluent to afford compound **2** as a colorless oil (4.78 g, 95%). The ^1H NMR spectrum of compound **2** is shown in Supplementary Fig. 8. ^1H NMR ($CDCl_3$, room temperature, 400 MHz) δ (ppm): 6.20 (t, J = 2.0 Hz, 2H), 4.10–4.04 (m, 2H), 4.03–3.94 (m, 2H), 3.07 (p, J = 1.6 Hz, 2H), 2.60 (d, J = 2.0 Hz, 2H), 2.13 (d, J = 9.0 Hz, 1H), 1.64–1.53 (m, 4H), 1.48 (d, J = 9.2 Hz, 1H), 1.42–1.29 (m, 4H), 0.92 (t, J = 7.2 Hz, 6H).

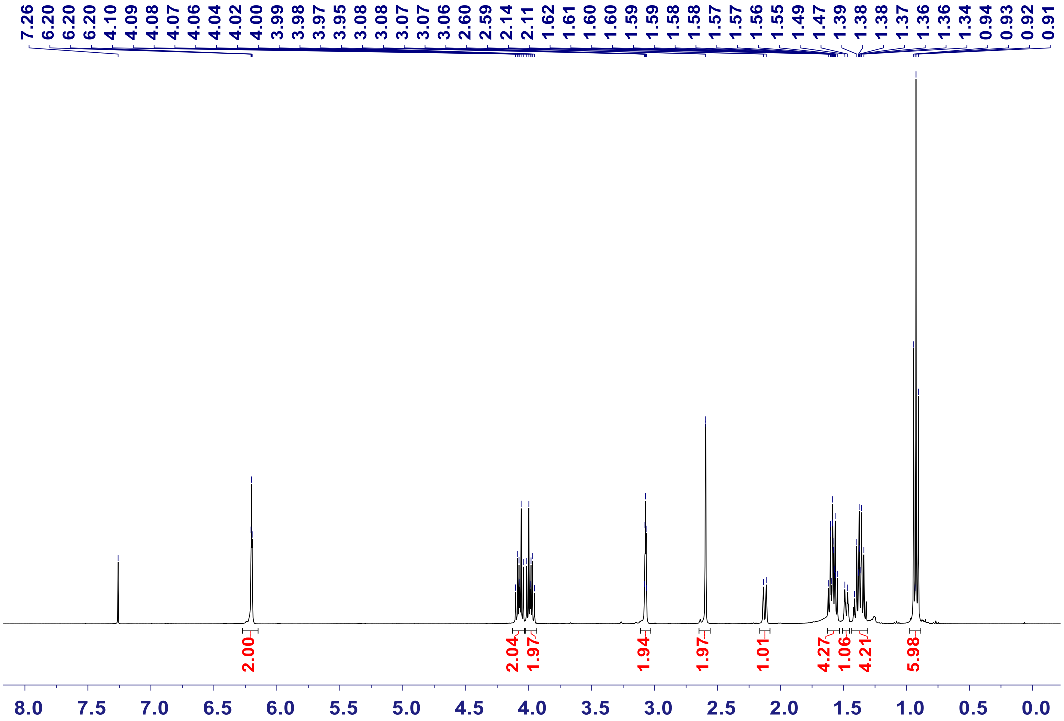

Supplementary Fig. 8 ^1H NMR spectrum ($CDCl_3$, room temperature, 400 MHz) of compound **2**.

Synthesis of compound 3 (ref. 2)

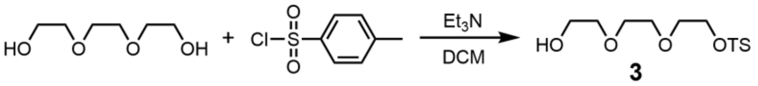

Triethylene glycol (60.0 g, 400 mmol) was dissolved in 60 mL of dry DCM under a nitrogen atmosphere. The solution was cooled to 0 °C with an ice bath and trimethylamine (12.1 g, 120 mmol) was added. Subsequently tosyl chloride (15.3 g, 80.0 mmol) was added in portions to the cooled reaction mixture. After complete addition, the solution was allowed to warm to room temperature and was stirred for 18 h. The mixture was washed with water, the aqueous fractions were re-extracted with DCM and the combined organic layers were washed with 5% citric acid, dried over Na_2SO_4, filtered and the solvent was removed under vacuum. The crude was further purified by gel chromatography with dichloromethane as an eluent to afford compound **3** as a colorless oil (20.7 g, 69%). The ^{1}H NMR spectrum of monomer **3** is shown in Supplementary Fig. 9. ^{1}H NMR ($CDCl_3$, room temperature, 400 MHz) δ (ppm): 7.80 (d, J = 8.4 Hz, 2H), 7.36–7.32 (m, 2H), 4.19–4.14 (m, 2H), 3.73–3.68 (m, 4H), 3.61 (s, 4H), 3.59–3.55 (m, 2H), 2.44 (s, 3H), 2.04 (s, 1H).

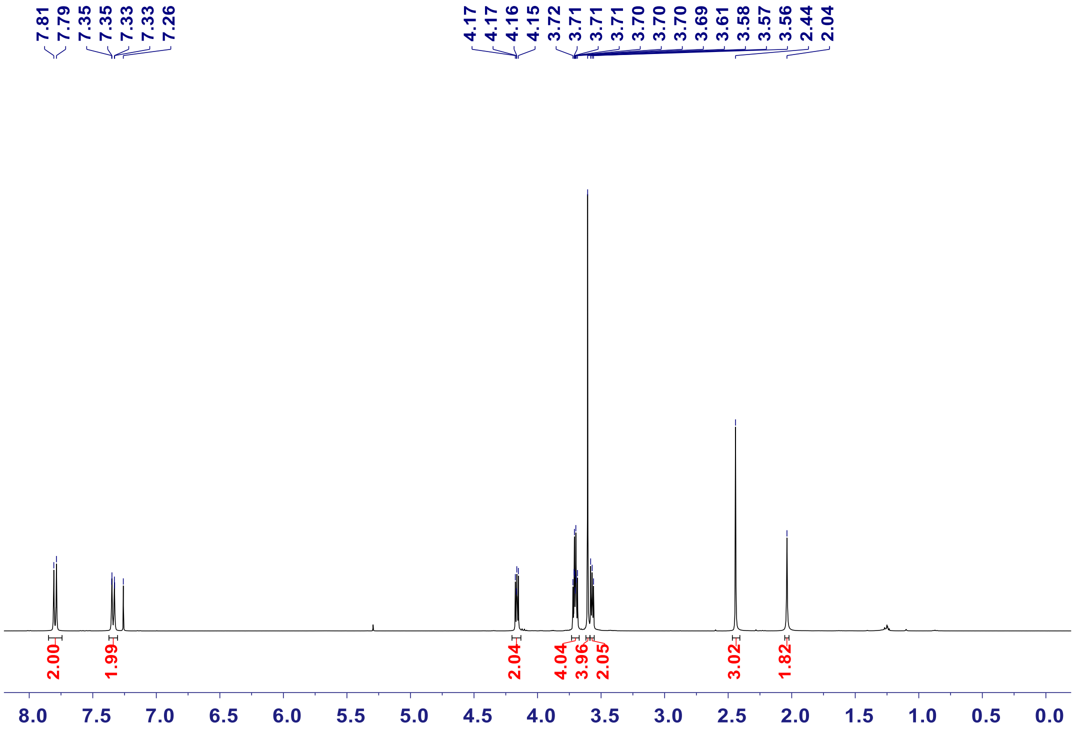

Supplementary Fig. 9 ^{1}H NMR spectrum ($CDCl_3$, room temperature, 400 MHz) of compound **3**.

Synthesis of compound 4 (ref. 2)

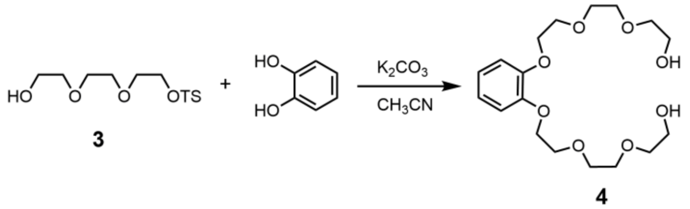

A slurry of catechol (2.83 g, 26.0 mmol), monotosylated glycol (16.0 g, 52.0 mmol) and K_2CO_3 (16.0 g, 120 mmol) in dry MeCN (200 mL) was refluxed under a N_2 atmosphere for 16 h. The reaction mixture was allowed to cool to room temperature, filtrated and removed the solvent under vacuum. The solid was re-dissolved in DCM and washed with H_2O. The organic layer was dried by $MgSO_4$, filtered and solvent removed under vacuum. The crude product was purified by column chromatography with ethyl acetate/methanol = (20/1 v/v) as an eluent to yield compound **4** as a colorless oil (6.50 g, 67%). The ^{1}H NMR spectrum of monomer **4** is shown in Supplementary Fig. 10. ^{1}H NMR ($CDCl_3$, room temperature, 400 MHz) δ (ppm): 6.90 (s, 4H), 4.18–4.14 (m, 4H), 3.89–3.84 (m, 4H), 3.76–3.65 (m, 12H), 3.61–3.57 (m, 4H), 3.22 (s, 2H).

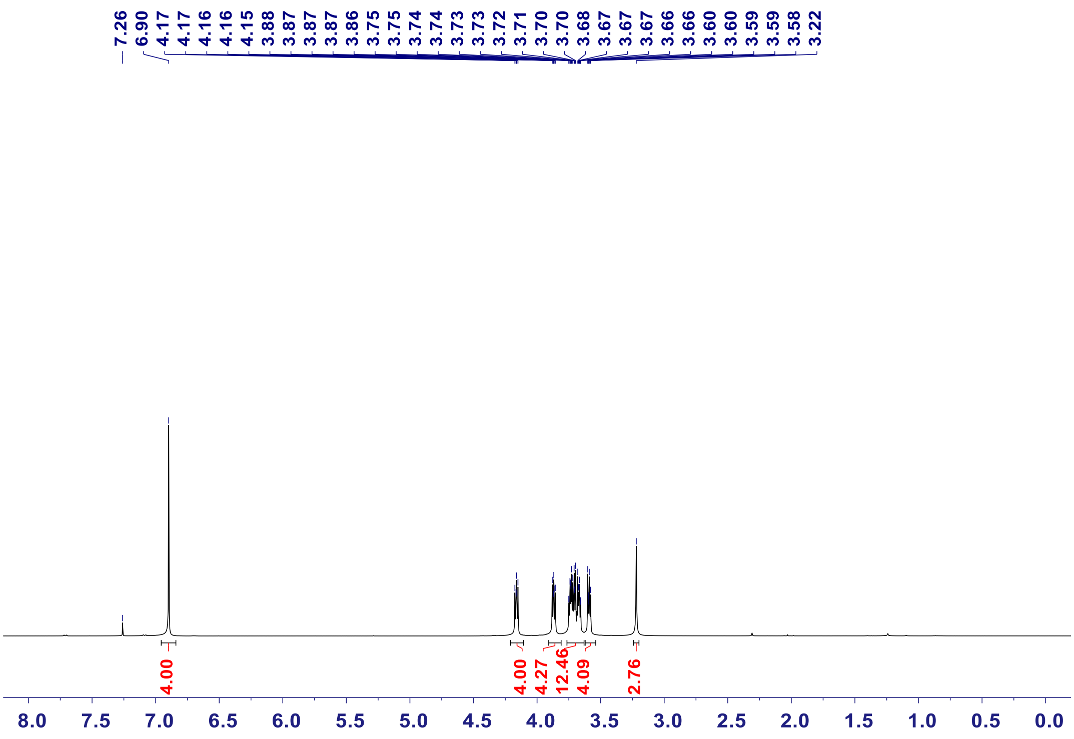

Supplementary Fig. 10 ^{1}H NMR spectrum ($CDCl_3$, room temperature, 400 MHz) of compound **4**.

Synthesis of compound 5 (ref. 2)

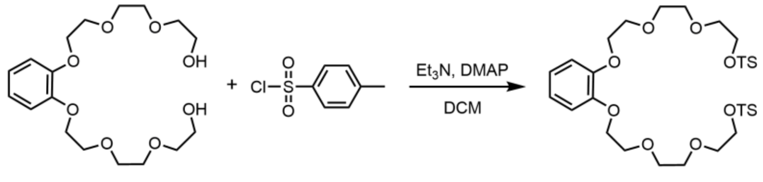

4 **5**

The compound **4** (6.00 g, 16.0 mmol), DMAP (400 mg, 3.20 mmol), and Et_3N (13.3 mL) were dissolved in CH_2Cl_2 (80 mL) and cooled to 0 °C. Tosyl chloride (12.2 g, 64.0 mmol) dissolved in CH_2Cl_2 (120 mL) was added dropwise to the cooled reaction mixture over a period of 3 h. The reaction mixture was allowed to reach room temperature and stirred for 19 h. The organic phase was washed with 1 M HCl, aqueous NaCl, and water. The organic phase was dried using Na_2SO_4 and the crude was further purified by gel chromatography with petroleum ether/ethyl acetate = (3/1 v/v) as an eluent to afford compound **5** as a yellow oil (8.60 g, 79%). The ^{1}H NMR spectrum of compound **5** is shown in Supplementary Fig. 11. ^{1}H NMR ($CDCl_3$, room temperature, 400 MHz) δ (ppm): 7.78 (d, J = 8.0 Hz, 4H), 7.31 (d, J = 8.0 Hz, 4H), 6.90 (s, 4H), 4.13 (q, J = 4.8 Hz, 8H), 3.81 (dd, J = 5.6, 4.4 Hz, 4H), 3.71–3.63 (m, 8H), 3.59 (dd, J = 5.6, 3.2 Hz, 4H), 2.42 (s, 6H).

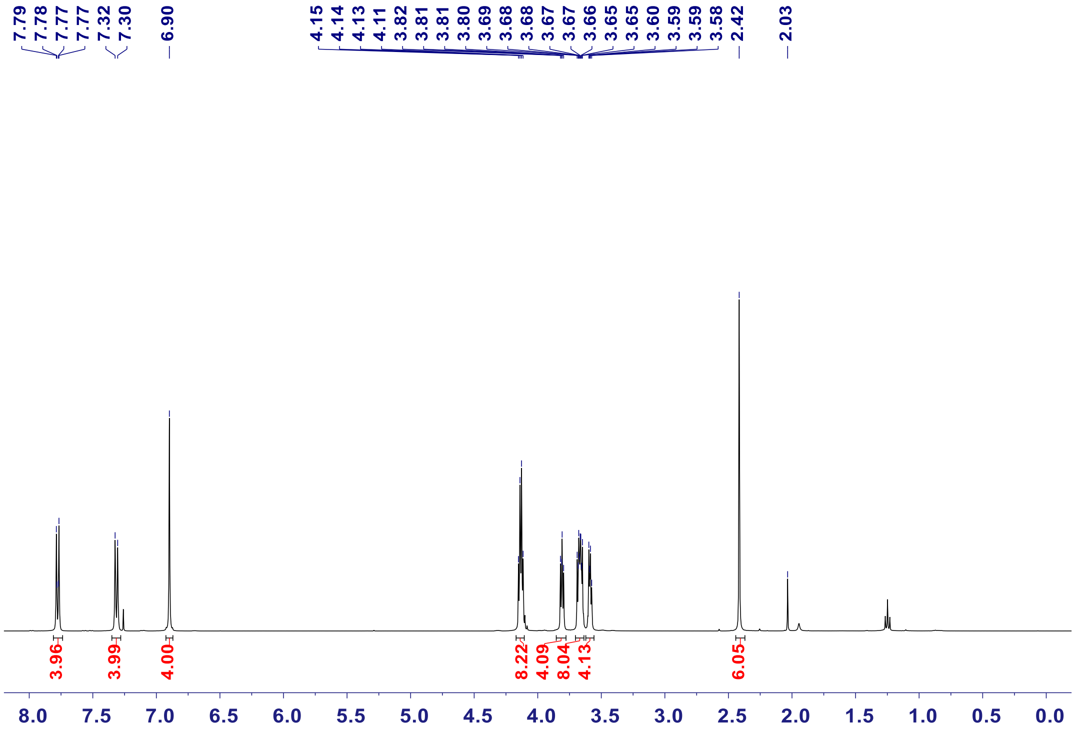

Supplementary Fig. 11 ^{1}H NMR spectrum ($CDCl_3$, room temperature, 400 MHz) of compound **5**.

Synthesis of compound 6 (ref. 2)

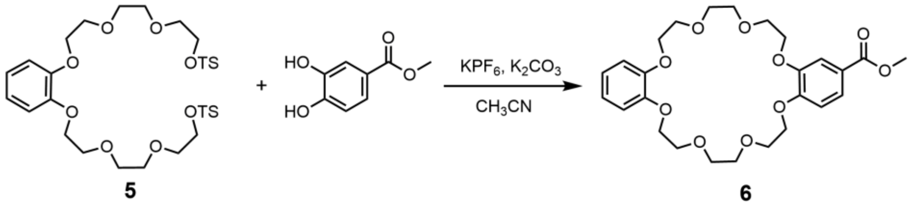

Compound **5** (8.50 g, 12.4 mmol), methyl 3,4-dihydroxybenzoate (2.10 g, 12.5 mmol) and KPF_6 (2.70 g, 14.7 mmol) were dissolved in MeCN (350 mL). The solution was degassed by bubbling N_2 through it, and K_2CO_3 (6.90 g, 50.0 mmol) was added. The mixture was heated at reflux under N_2 for 3 days, allowed to cool to room temperature and the solids were filtered. The solvent was removed by rotary evaporation and the residue was dissolved in chloroform. The solution was washed with saturated NaCl solution. The organic layer was dried by Na_2SO_4, and the crude was further purified by gel chromatography with dichloromethane/methanol = (100/1 v/v) as an eluent to afford compound **6** as a yellow oil (4.65 g, 74%). The ^{1}H NMR spectrum of compound **6** is shown in Supplementary Fig. 12. ^{1}H NMR ($CDCl_3$, room temperature, 400 MHz) δ (ppm): 7.63 (dd, J = 8.4, 2.0 Hz, 1H), 7.51 (d, J = 2.0 Hz, 2H), 6.91–6.81 (m, 5H), 4.20–4.16 (m, 4H), 4.15–4.11 (m, 4H), 3.95–3.88 (m, 8H), 3.86 (s, 3H), 3.87–3.83 (m, 8H).

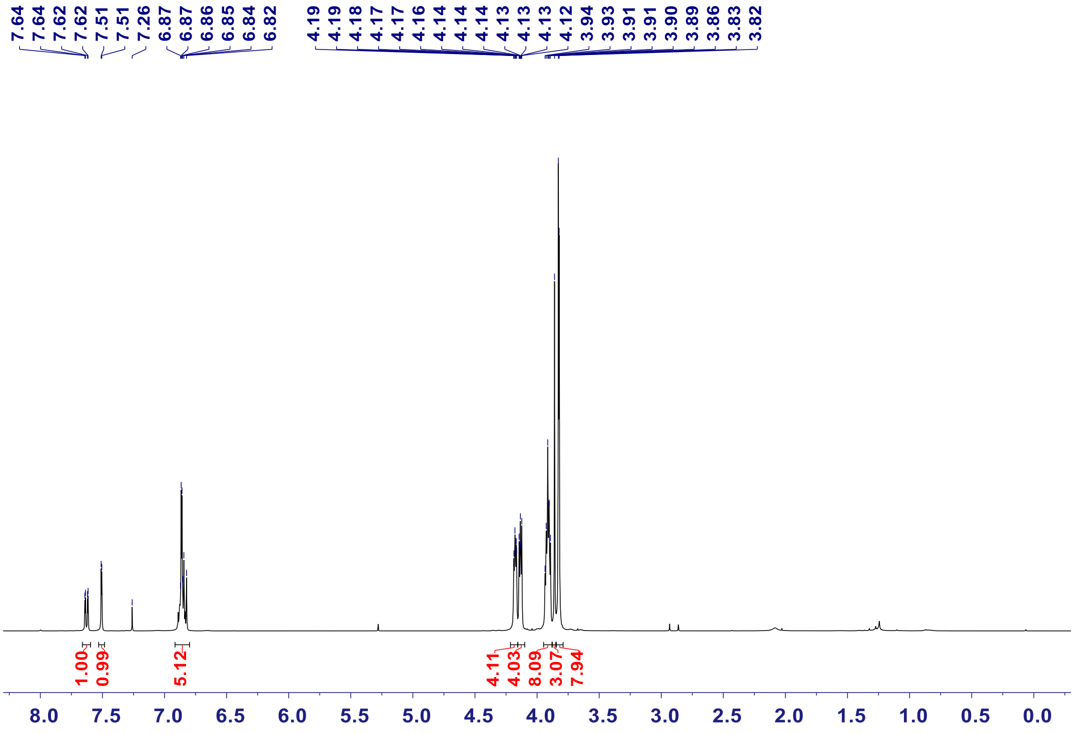

Supplementary Fig. 12 ^{1}H NMR spectrum ($CDCl_3$, room temperature, 400 MHz) of compound **6**.

Synthesis of compound 7 (ref. 2)

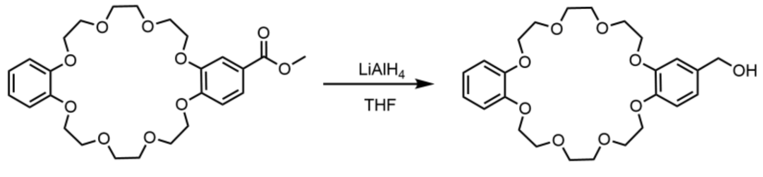

To a solution of compound **6** (4.56 g, 9.00 mmol) in THF (200 mL) at 0 °C was added portionwise lithium aluminum hydride (1.02 g, 26.9 mmol), then the solution was heated to reflux for 2 h. After cooling to room temperature, the mixture was treated by adding water (1.2 mL) cautiously, and then 15% NaOH solution (1.2 mL) was added. The precipitate was filtered and the crude was further purified by gel chromatography with dichloromethane/methanol = (10/1 v/v) as an eluent to afford compound **7** as a yellow oil (3.70 g, 86%). The ^{1}H NMR spectrum of compound **7** is shown in Supplementary Fig. 13. ^{1}H NMR ($CDCl_3$, room temperature, 400 MHz) δ (ppm): 6.93–6.80 (m, 7H), 4.58 (s, 2H), 4.19–4.11 (m, 8H), 3.91 (dd, J = 5.2, 3.6 Hz, 8H), 3.87–3.83 (m, 8H).

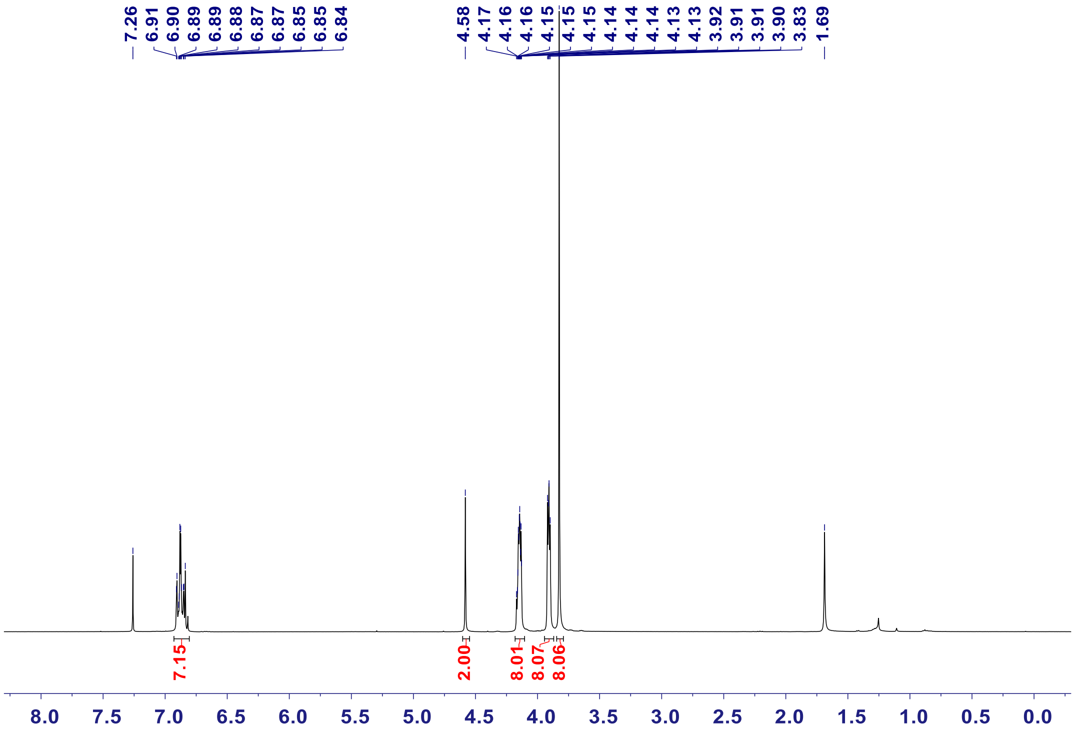

Supplementary Fig. 13 ^{1}H NMR spectrum ($CDCl_3$, room temperature, 400 MHz) of compound **7**.

Synthesis of compound 8

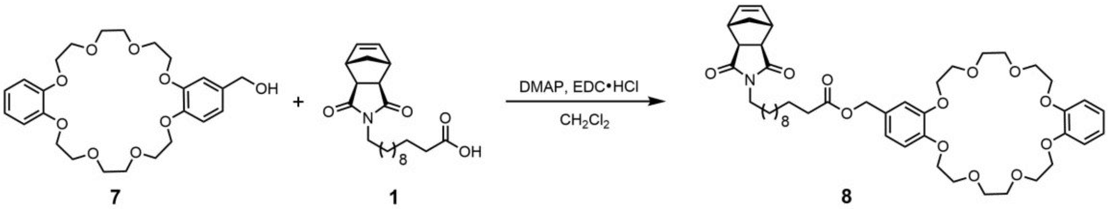

Compound **7** (1.91 g, 4.00 mmol), EDC·HCl (0.92 g, 4.80 mmol), and DMAP (0.24 g, 1.96 mmol) were dissolved in 40 mL of DCM. Compound **1** (1.68 g, 4.83 mmol) was added. The mixture was stirred at room temperature overnight and purified via gel chromatography with dichloromethane/ethyl acetate = (100/1 v/v) as an eluent to afford compound **8** as a white solid (2.52 g, 78%). M.p. = 44 °C. The ^{1}H NMR spectrum of compound **8** is shown in Supplementary Fig. 14. ^{1}H NMR ($CDCl_3$, room temperature, 400 MHz): 6.92–6.78 (m, 7H), 6.27 (t, *J* = 1.6 Hz, 2H), 4.99 (s, 2H), 4.19–4.08 (m, 8H), 3.93–3.87 (m, 8H), 3.84 (s, 8H), 3.46–3.40 (m, 2H), 3.26 (q, *J* = 1.6 Hz, 2H), 2.65 (d, *J* = 1.2 Hz, 2H), 2.30 (dd, *J* = 9.2, 6.0 Hz, 2H), 1.66–1.46 (m, 6H), 1.35–1.17 (m, 12H). The ^{13}C NMR spectrum of compound **8** is shown in Supplementary Fig. 15. ^{13}C NMR ($CDCl_3$, room temperature, 100 MHz) δ (ppm): 178.11, 173.72, 148.90, 137.82, 129.15, 121.65, 121.40, 114.29, 114.02, 113.59, 71.29, 69.93, 69.37, 66.03, 47.79, 45.16, 42.71, 38.74, 34.35, 29.36, 29.32, 29.19, 29.09, 27.76, 26.94, 24.92. HRESIMS is shown in Supplementary Fig. 16: *m*/*z* calcd for $C_{20}H_{29}NO_4$, 830.4085 $[M + H]^+$; found 830.4086 $[M + H]^+$.

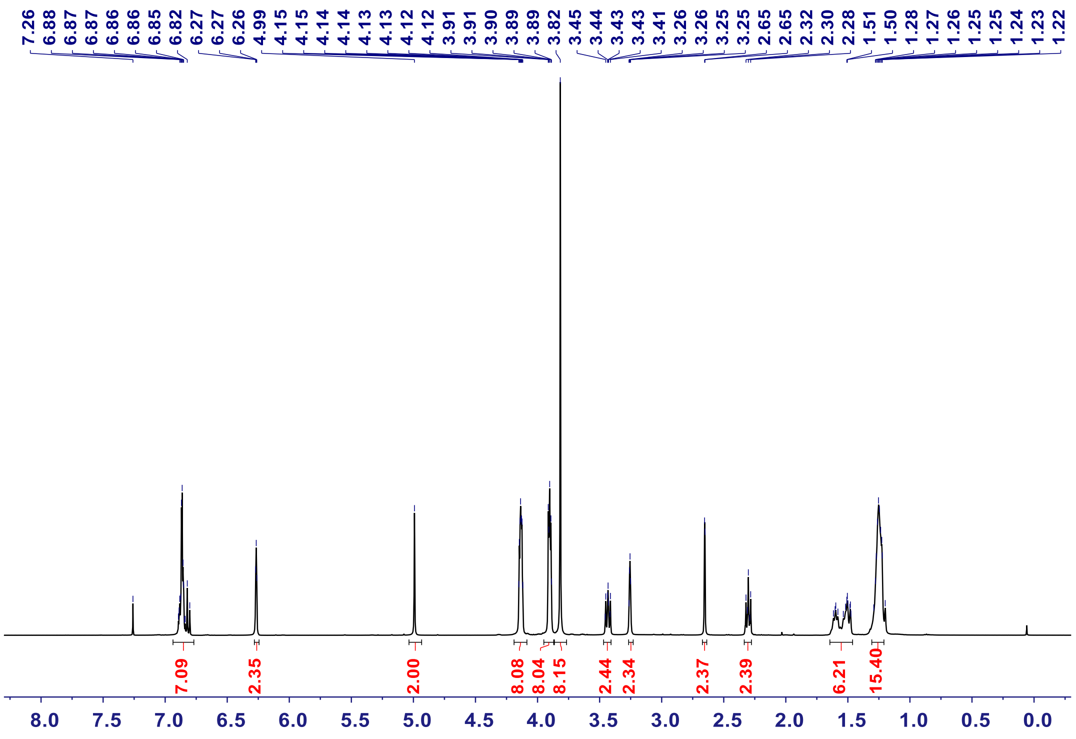

Supplementary Fig. 14 ^{1}H NMR spectrum ($CDCl_3$, room temperature, 400 MHz) of compound **8**.

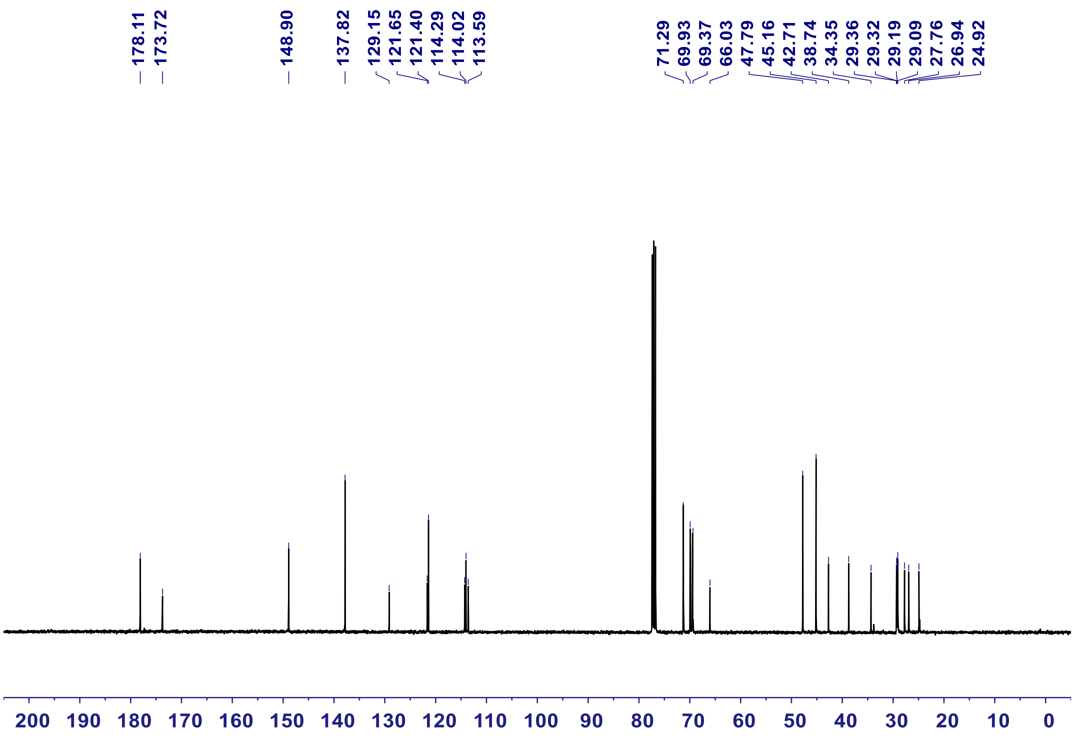

Supplementary Fig. 15 ^{13}C NMR spectrum ($CDCl_3$, room temperature, 100 MHz) of compound **8**.

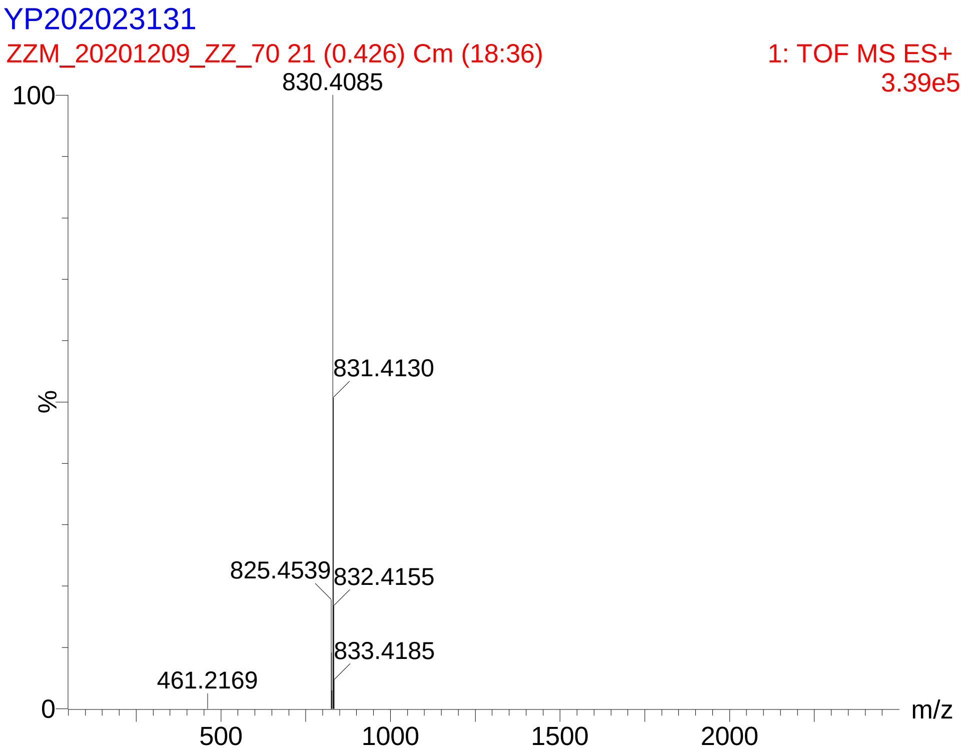

Supplementary Fig. 16 Electrospray ionization mass spectrum of compound **8**.

Synthesis of covalent polymer

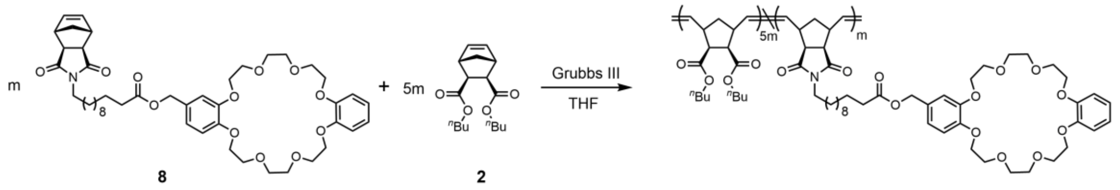

An oven-dried vial was charged with compound **8** (566 mg, 0.701 mmol), compound **2** (1.03 g, 3.50 mmol) and a stir bar. The vial was then degassed, and the 40 mL of degassed anhydrous THF was added via syringe under a nitrogen atmosphere to dissolve the monomer. The Ru catalyst (17.6 mg, 19.9 μmol) in 2 mL degassed anhydrous THF was injected into the monomer solution to initiate the polymerization. The solution was stirred for 16 h at room temperature and then quenched by the addition of 0.1 mL of neat ethyl vinyl ether. After stirring for an additional 60 min, the mixture was added dropwise to 400 mL of rapidly stirring methanol and a light green precipitate formed immediately. The suspension was then centrifuged and collected. The solid was dried under vacuum for 24 h to afford the target polymer (1.32g, 83%). The ^{1}H NMR spectrum of the polymer is shown in Supplementary Fig. 17. The SEC curve of the polymer is shown in Supplementary Fig. 18 with M_n = 7.7 kDa.

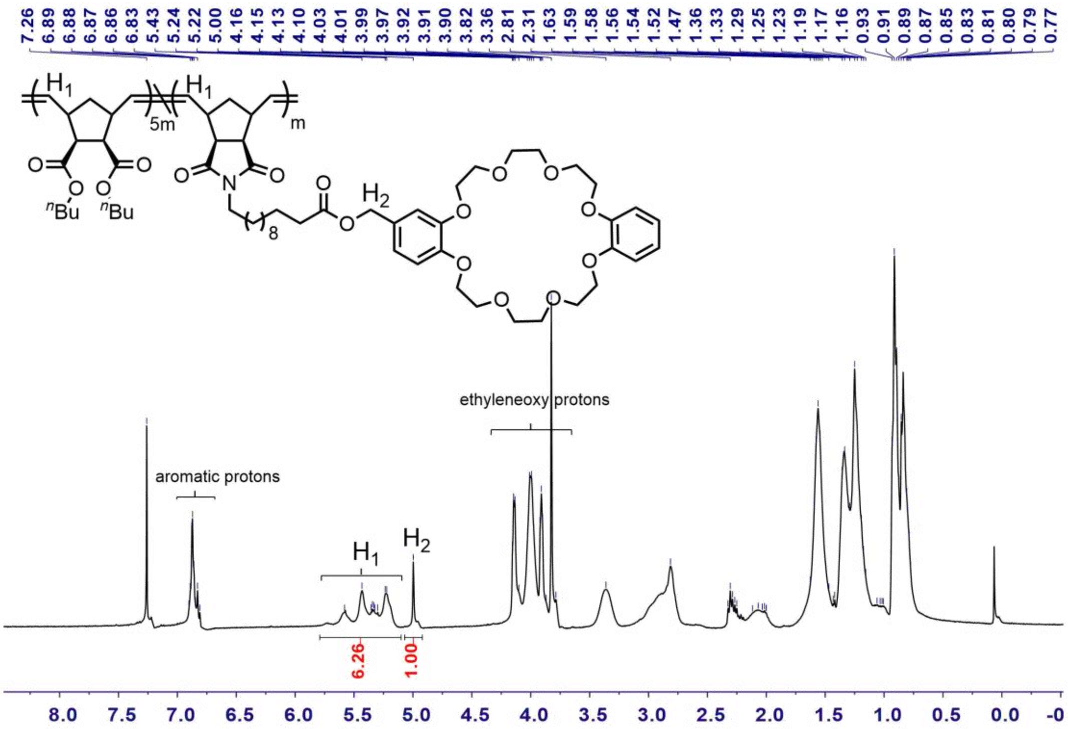

Supplementary Fig. 17 ^{1}H NMR spectrum ($CDCl_3$, room temperature, 400 MHz) of the DB24C8-based polymer.

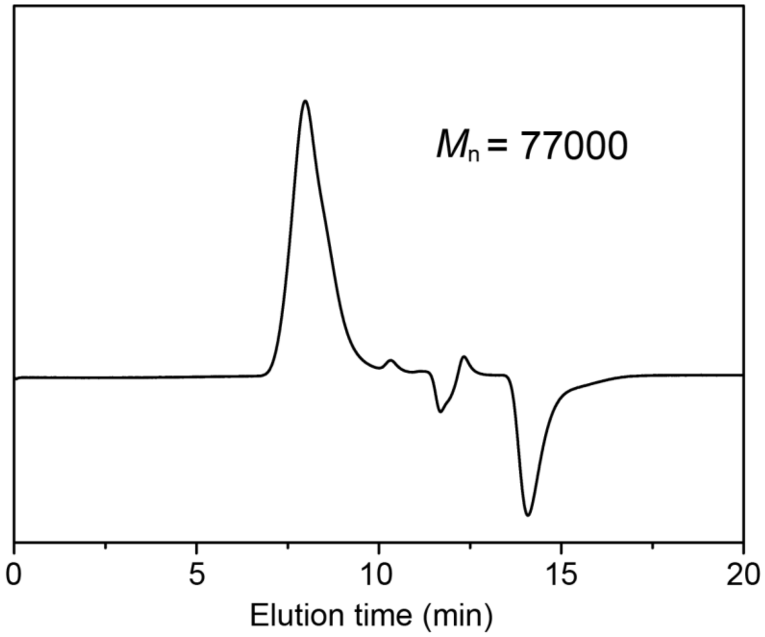

Supplementary Fig. 18 GPC elution curve of the DB24C8-based polymer with DMF as the eluent and PS as the standard.

Synthesis of compound 9 (ref. 3)

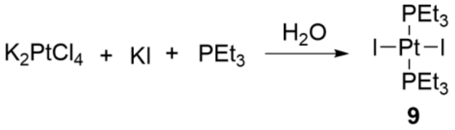

A K_2PtCl_4 (2.00 g, 4.82 mmol) was dissolved in deoxygenated distilled water (30 mL) in a two necked round-bottomed flask, sealed with two septa, filled with argon, and vigorously stirred. To this a solution of KI (1.60 g, 9.64 mmol) in deoxygenated water (7 mL) was added, immediately followed by the addition of PEt_3 (1.47 mL, 9.65 mmol). The reaction mixture was vigorously stirred at room temperature under a constant argon flow until the originally red solution became colorless. The resulting yellow precipitate was filtered off, washed with distilled water, and air-dried. The crude was further purified via gel chromatography with petroleum ether/dichloromethane = (100/1 v/v) as an eluent to afford **9** as a bright yellow solid (2.80 g, 85%). The ^1H NMR spectrum of the polymer is shown in Supplementary Fig. 19. ^1H NMR ($CDCl_3$, room temperature, 400 MHz) δ (ppm): 2.35–2.20 (m, 12H), 1.16–1.02 (m, 18H).

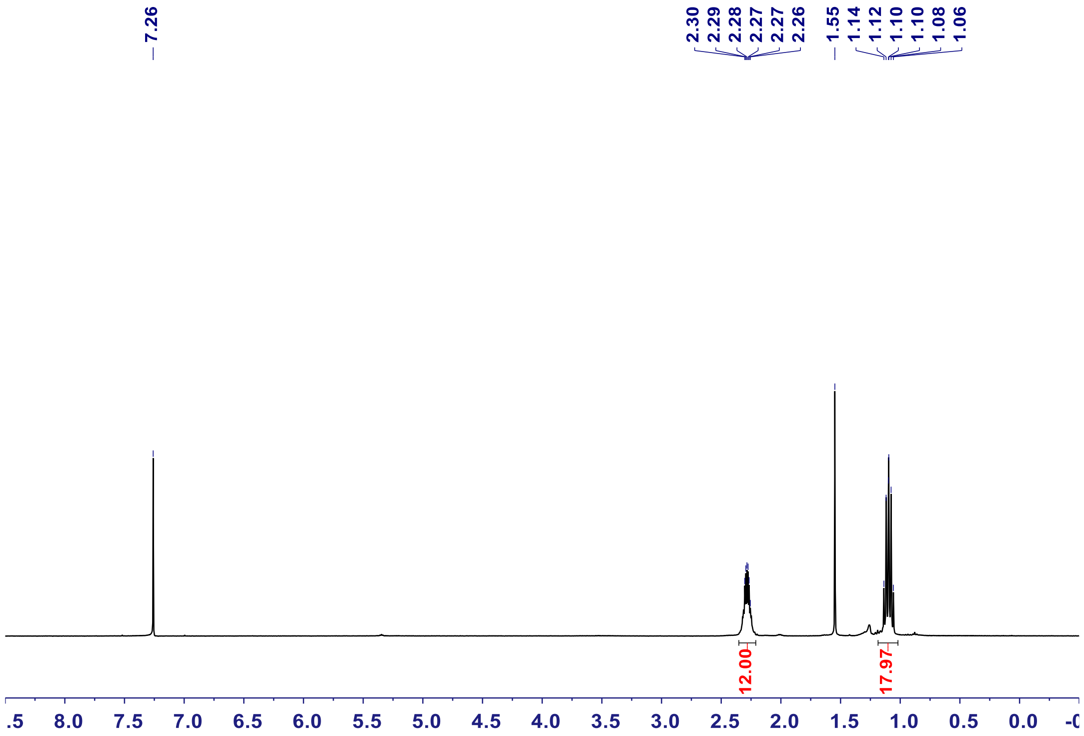

Supplementary Fig. 19 ^1H NMR spectrum ($CDCl_3$, room temperature, 400 MHz) of compound **9**.

Synthesis of compound 10 (ref. 4)

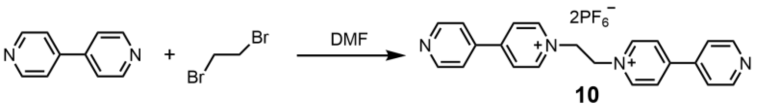

A mixture of 4,4'-bipyridine (4.99 g, 32.0 mmol) and 1,2-dibromoethane (2.00 g, 10.7 mmol) was dissolved in DMF and heated at 70 °C for 12 h. A pale yellow precipitate separated out and filtered, then recrystallized in water and filtered. The residue was dissolved in hot water and a saturated aqueous NH_4PF_6 solution was added to produce a precipitate, which was collected by vacuum filtration to afford **10** (1.34 g, 20%) as a pale purple solid. The ^{1}H NMR spectrum of **10** is shown in Supplementary Fig. 20. ^{1}H NMR (acetone-d_6, room temperature, 400 MHz) δ (ppm): 9.39 (d, J = 6.8 Hz, 4H), 8.89 (d, J = 6.0 Hz, 4H), 8.79 (d, J = 7.2 Hz, 4H), 7.99 (d, J = 6.0 Hz, 4H), 5.84 (s, 4H).

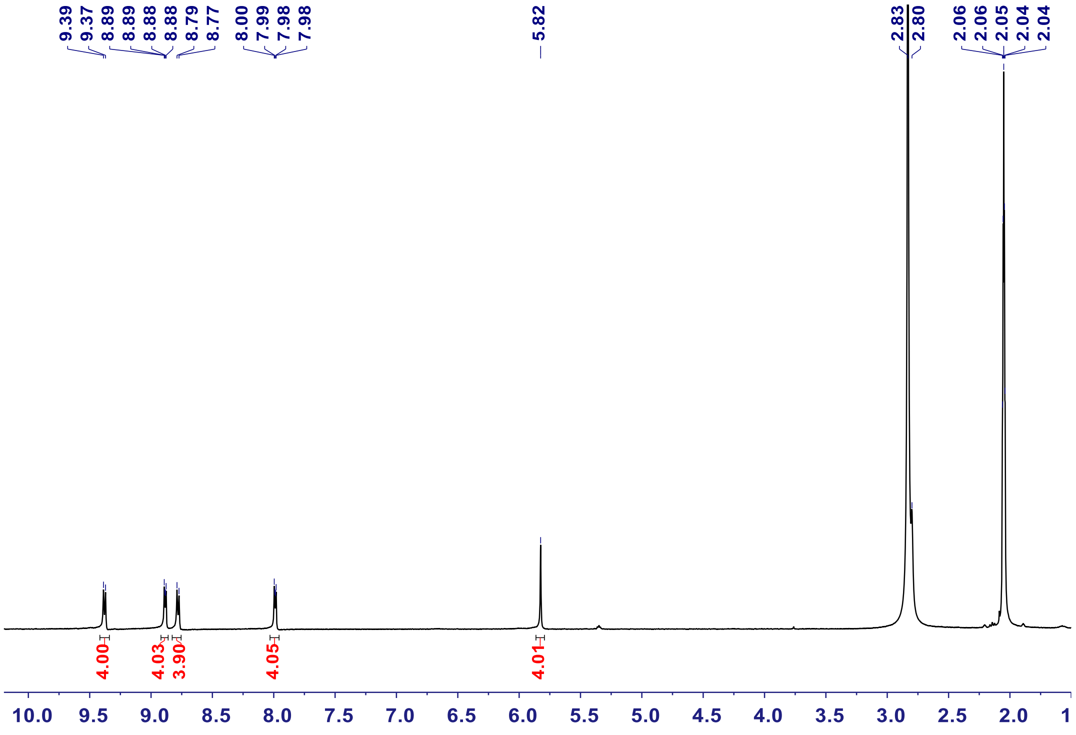

Supplementary Fig. 20 ^{1}H NMR spectrum (acetone-d_6, room temperature, 400 MHz) of compound **10**.

Synthesis of compound 11 (ref. 5)

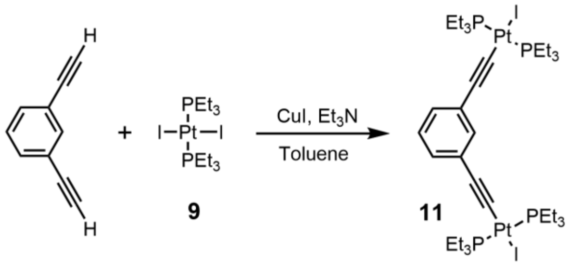

To a 100 mL round-bottom Schlenk flask were added 1,3-diethynylbenzene (111 mg, 0.88 mmol) and **9** (2.42 g, 3.53 mmol). Then 40 mL of toluene and 20 mL of dry ethylamine were added under nitrogen. The solution was stirred for 10 min at room temperature before CuI (40.0 mg, 0.21 mmol) was added in one portion. After 16 h at room temperature, a small amount of diethylammonium iodide started precipitating out of solution. The solvent was removed in vacuo, and the crude was further purified by gel chromatography with petroleum ether/dichloromethane = (3/1 v/v) as an eluent to afford **11** as a pale green solid (786 mg, 72%). The ^{1}H NMR spectrum of **11** is shown in Supplementary Fig. 21. ^{1}H NMR ($CDCl_3$, room temperature, 400 MHz) δ (ppm): 7.20 (s, 1H), 7.09 (s, 3H), 2.30–2.15 (m, 24H), 1.23–1.10 (m, 36H). The ^{31}P{^{1}H} NMR spectrum of **11** is shown in Supplementary Fig. 22. ^{31}P{^{1}H} NMR spectrum ($CDCl_3$, room temperature, 161.8 MHz) δ (ppm): 8.45 (s, ^{195}Pt satellites, $^{1}J_{Pt-P}$ = 2324.13 Hz).

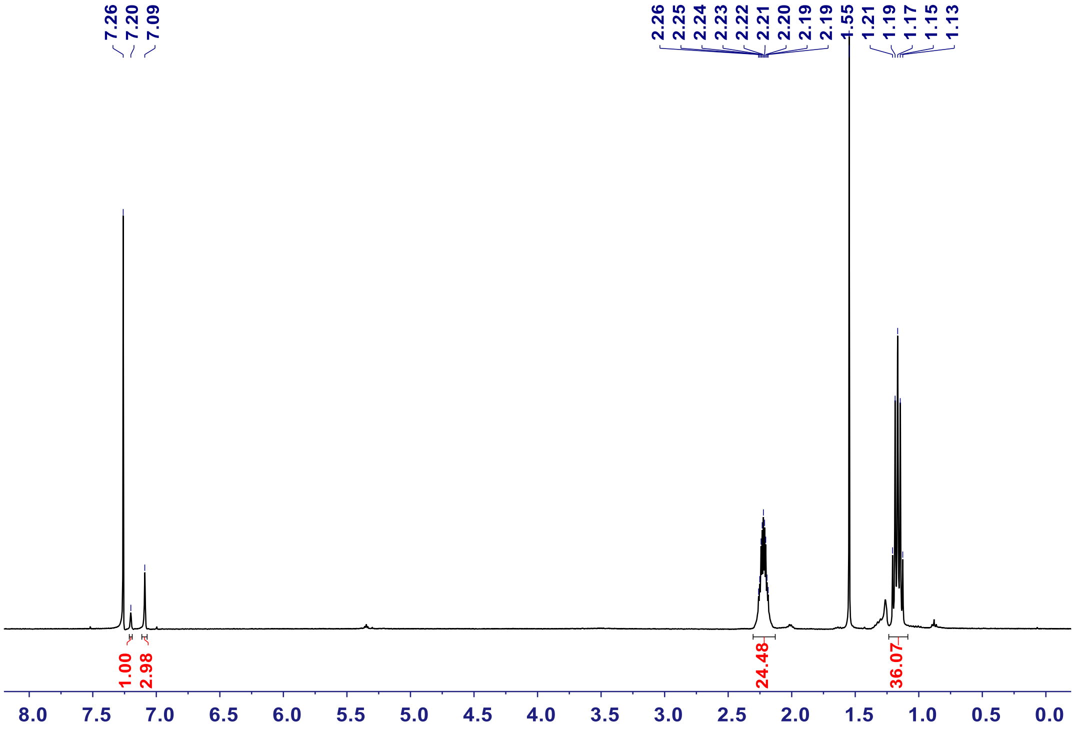

Supplementary Fig. 21 ^{1}H NMR spectrum ($CDCl_3$, room temperature, 400 MHz) of compound **11**.

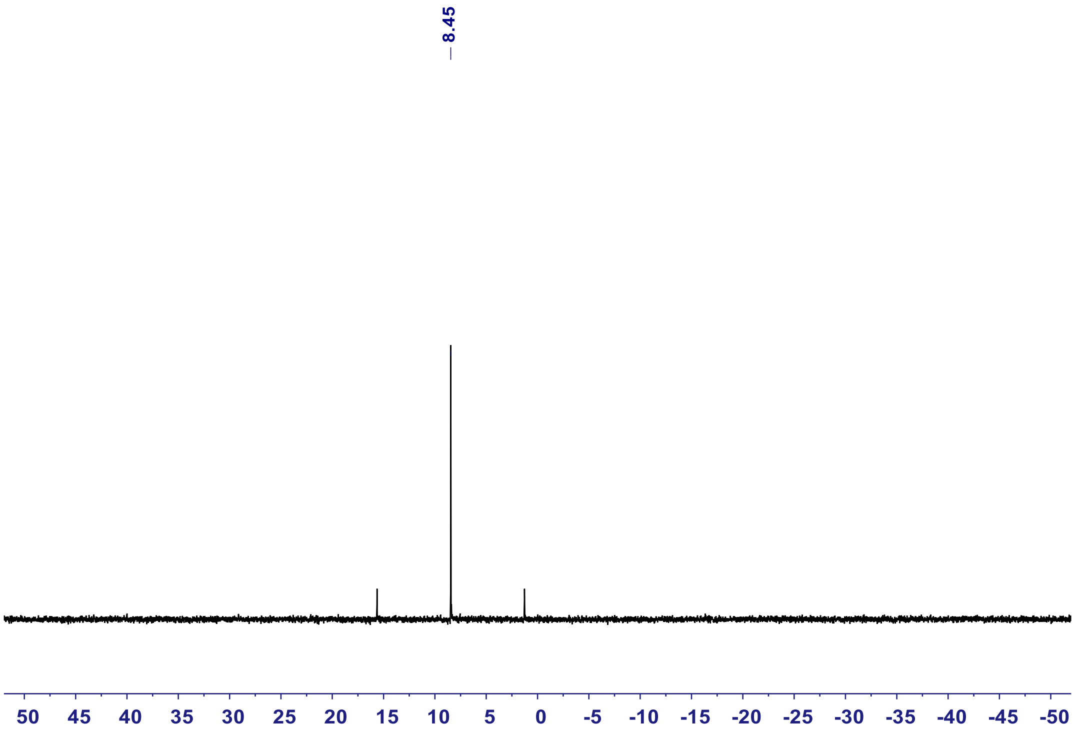

Supplementary Fig. 22 $^{31}P\{^{1}H\}$ NMR spectrum ($CDCl_3$, room temperature, 161.8 MHz) of compound **11**.

Synthesis of compound 12 (ref. 5)

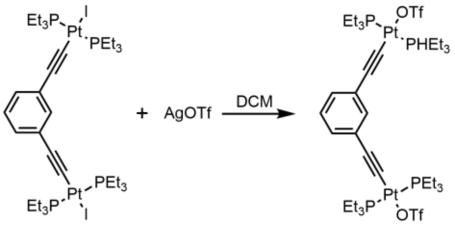

A 100 mL round-bottom Schlenk flask was charged with **11** (425 mg, 0.343 mmol) and 40 mL of dichloromethane. To the solution was added AgOTf (299 mg, 1.16 mmol) at once, resulting in a yellowish precipitate of AgI. After stirring for 4 h at room temperature, the suspension was filtered through a glass fiber and the volume of the solution was reduced to 5.0 mL. Subsequent addition of diethyl ether resulted in the precipitation of **12** as a slightly yellow powder (383 mg, 87%). The ^{1}H NMR spectrum of **12** is shown in Supplementary Fig. 23. ^{1}H NMR ($CDCl_3$, room temperature, 400 MHz) δ (ppm): 7.20 (s, 1H), 7.09 (s, 3H), 2.16–1.97 (m, 24H), 1.36–1.09 (m, 36H). The ^{31}P{^{1}H} NMR spectrum of **12** is shown in Supplementary Fig. 24. ^{31}P{^{1}H} NMR spectrum ($CDCl_3$, room temperature, 161.8 MHz) δ (ppm): 21.8 (s, ^{195}Pt satellites, $^{1}J_{Pt\text{–}P}$ = 2299.18 Hz).

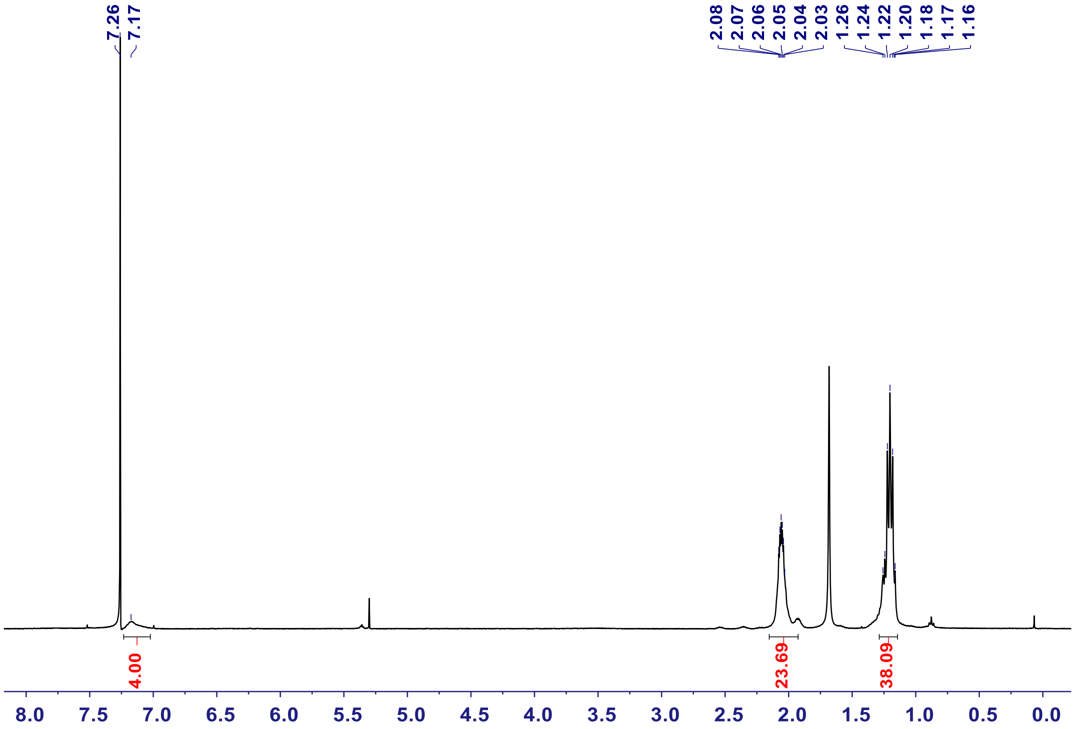

Supplementary Fig. 23 ^{1}H NMR spectrum ($CDCl_3$, room temperature, 400 MHz) of compound **12**.

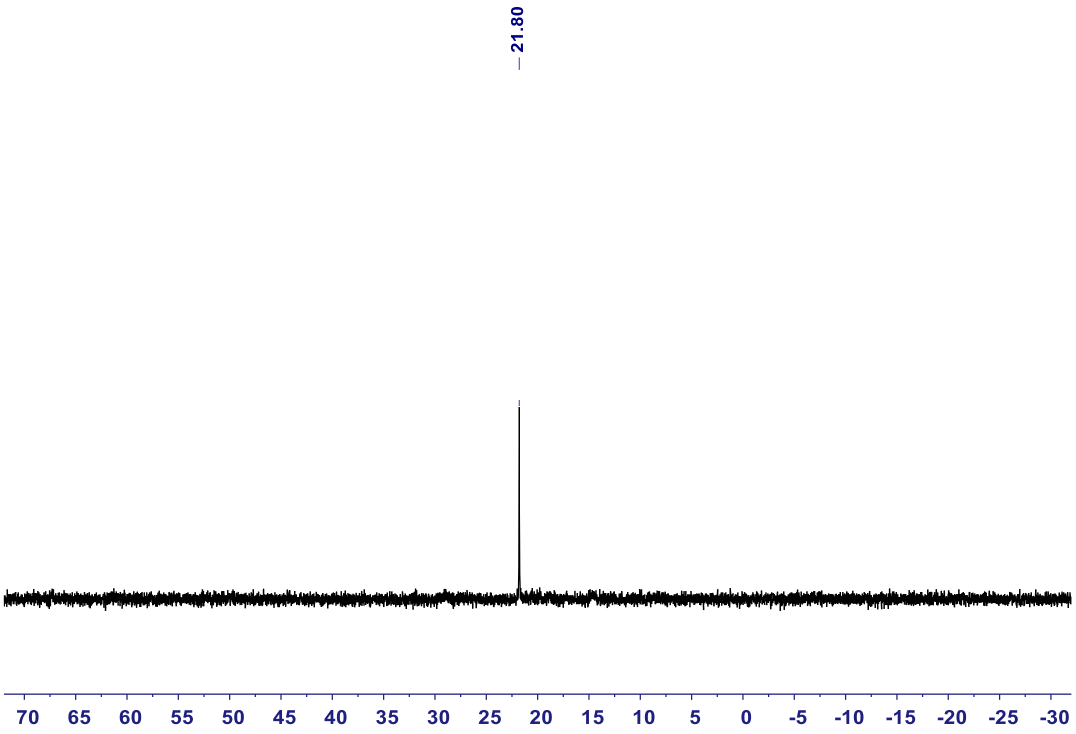

Supplementary Fig. 24 $^{31}P\{^{1}H\}$ NMR spectrum ($CDCl_3$, room temperature, 161.8 MHz) of compound **12**.

Synthesis of complex 13

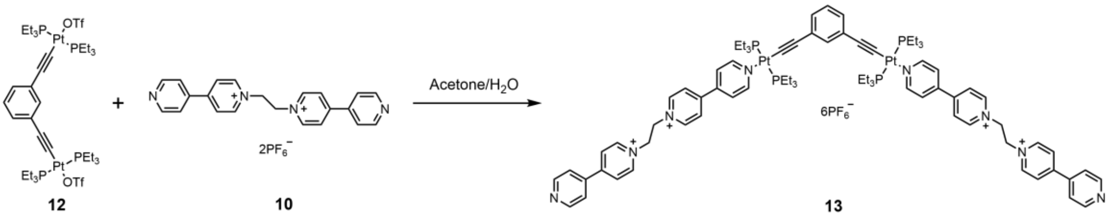

120° Diplatinum acceptor **12** (60.0 mg, 0.047 mmol) and 1,2-bis([4,4'-bipyridin]-1-ium)ethane ligand **10** (58.8 mg, 0.093 mmol) were placed in a vial, followed by addition of water (0.25 mL) and acetone (1.0 mL). After 5 h of stirring at room temperature, KPF_6 solution was added into the reaction solution. Acetone was removed by evaporation under vacuum. The resulted precipitate was collected by filtration and dried under vacuum to afford the complex as a pale yellow solid (923 mg, 94%). The ^{1}H NMR spectrum of metallacycle is shown in Supplementary Fig. 25. ^{1}H NMR (acetone-d_6, room temperature, 400 MHz) δ (ppm): 9.38–9.31 (m, 4 H), 9.31–8.19 (m, 8 H), 8.95–8.87 (m, 4 H), 8.80–8.70 (m, 8 H), 8.37–8.30 (m, 4 H), 8.08–8.00 (m, 4 H), 7.29–7.14 (m, 4 H), 5.89–5.73 (m, 8 H), 2.03–1.85 (m, 24 H), 1.36–1.16 (m, 36 H). The ^{31}P{^{1}H} NMR spectrum of metallacycle is shown in Supplementary Fig. 26. ^{31}P{^{1}H} NMR spectrum (acetone-d_6, room temperature, 161.8 MHz) δ (ppm): 15.4 (s, ^{195}Pt satellites, $^{1}J_{Pt\text{–}P}$ = 2306.33 Hz).

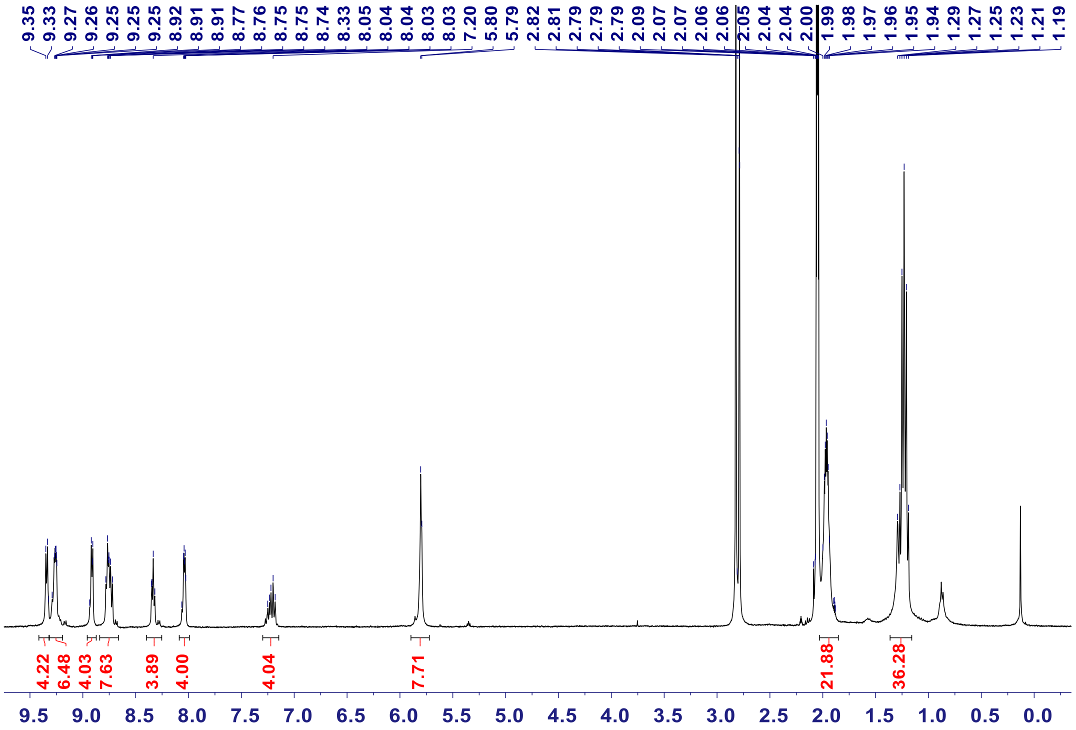

Supplementary Fig. 25 ^{1}H NMR spectrum (acetone-d_6, room temperature, 400 MHz) of complex **13**.

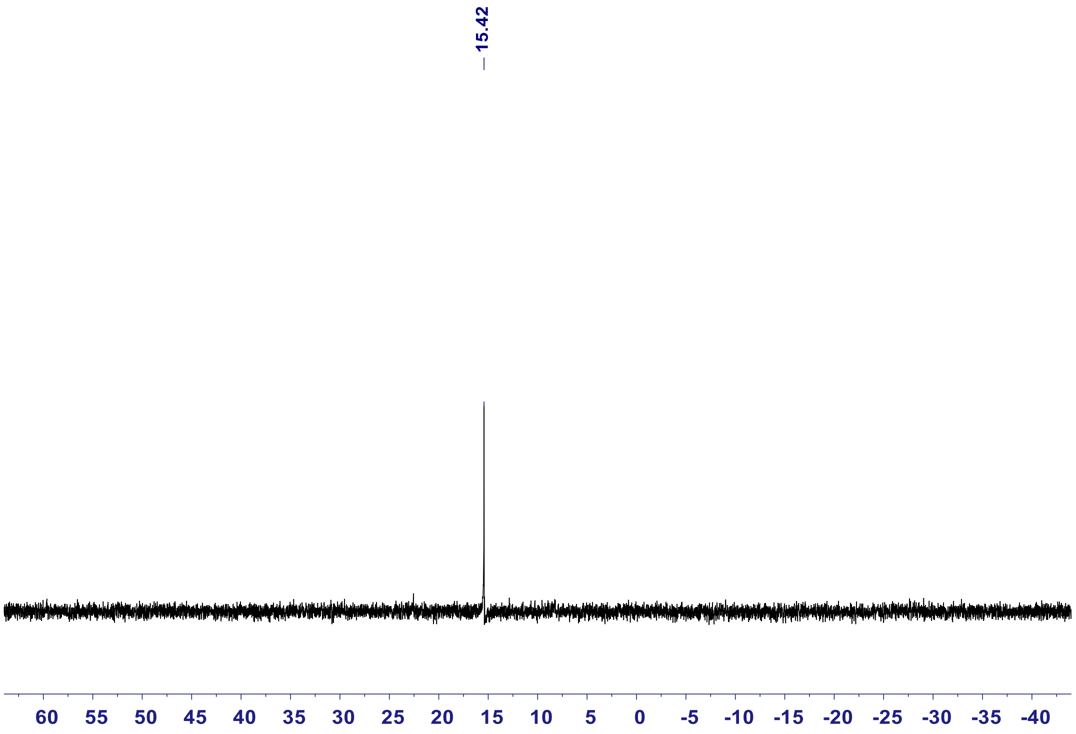

Supplementary Fig. 26 ^{31}P{^{1}H} NMR spectrum (acetone-d_6, room temperature, 161.8 MHz) of complex **13**.

Synthesis of metallacycle (ref. 6)

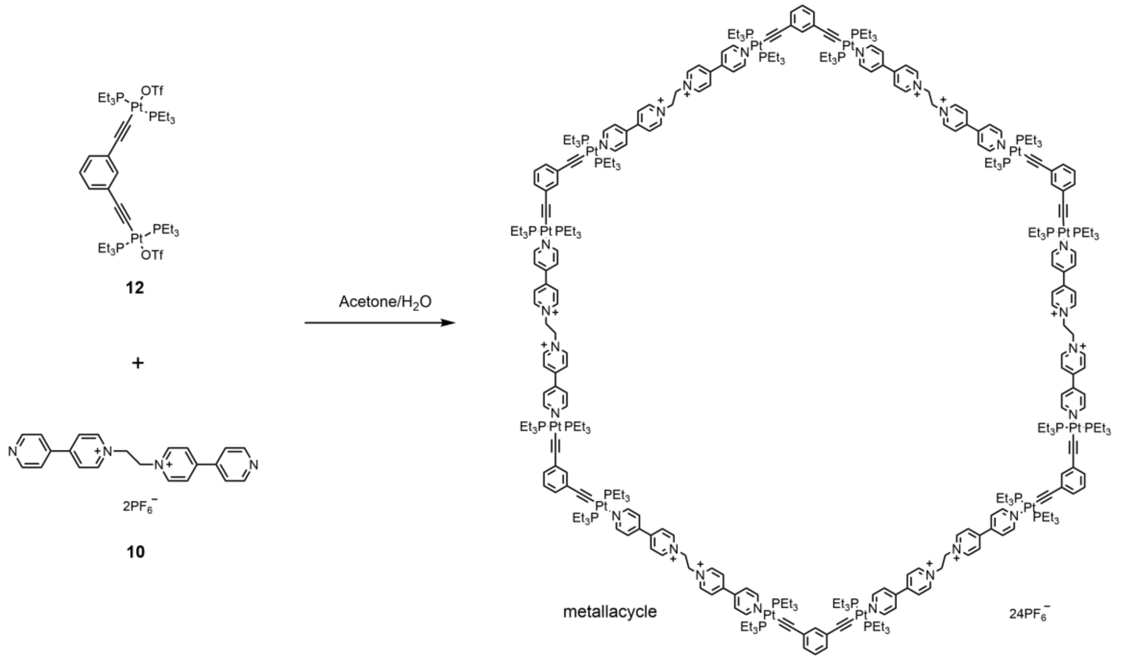

120° Diplatinum acceptor **12** (363 mg, 0.282 mmol) and 1,2-bis([4,4'-bipyridin]-1-ium)ethane ligand **10** (178 mg, 0.282 mmol) were placed in a vial, followed by addition of water (10 mL) and acetone (40 mL). After 5 h of stirring at room temperature, KPF_6 solution was added into the reaction solution. Acetone was removed by evaporation under vacuum. The resulted precipitate was collected by filtration and the solid was then re-dissolved in acetone (50 mL). Water (50 mL) was added into the solution and acetone was removed by evaporation under vacuum. The resulted precipitate was collected by filtration and dried under vacuum to afford the metallacycle as a light brown solid (538 mg, 81%). The ^{1}H NMR spectrum of metallacycle is shown in Supplementary Fig. 27. ^{1}H NMR (acetone-d_6, room temperature, 400 MHz) δ (ppm): 9.30–9.13 (m, 48 H), 8.80–8.66 (m, 24 H), 8.39–8.24 (m, 24 H), 7.31–7.13 (m, 24 H), 5.92–5.76 (m, 24 H), 1.98–1.86 (m, 144 H), 1.40–1.15 (m, 216 H). The $^{31}P\{^{1}H\}$ NMR spectrum of metallacycle is shown in Supplementary Fig. 28. $^{31}P\{^{1}H\}$ NMR spectrum (acetone-d_6, room temperature, 161.8 MHz) δ (ppm): 15.4 (s, ^{195}Pt satellites, $^{1}J_{Pt–P}$ = 2306.33 Hz).

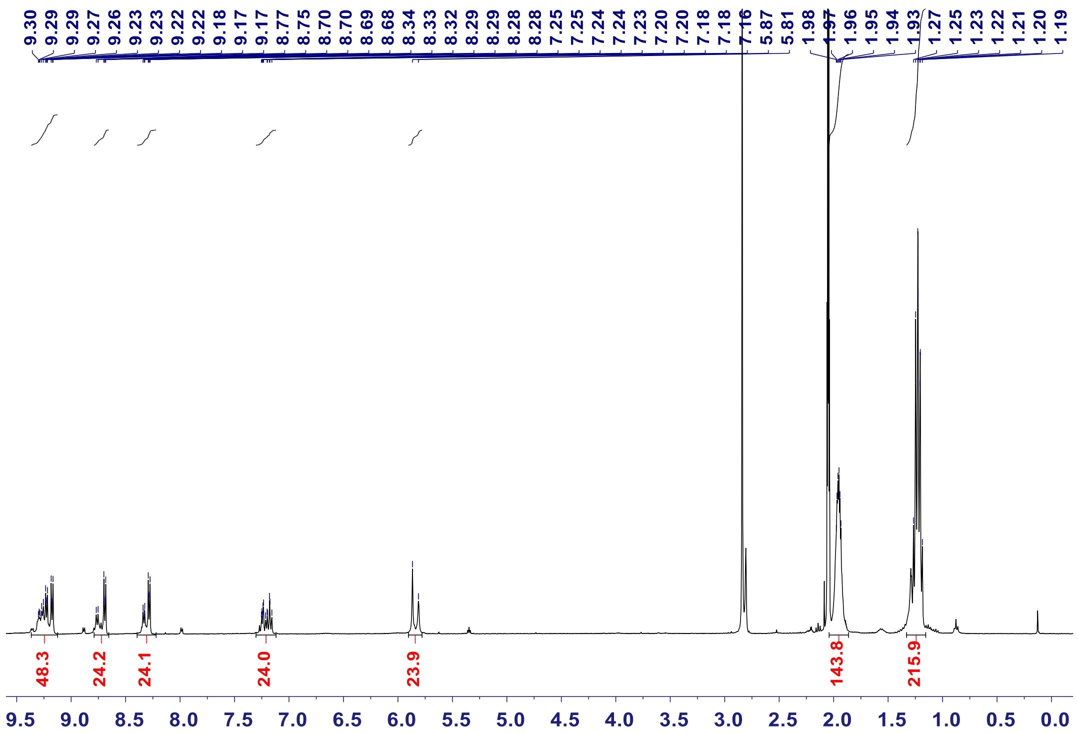

Supplementary Fig. 27 ^{1}H NMR spectrum (acetone-d_6, room temperature, 400 MHz) of metallacycle.

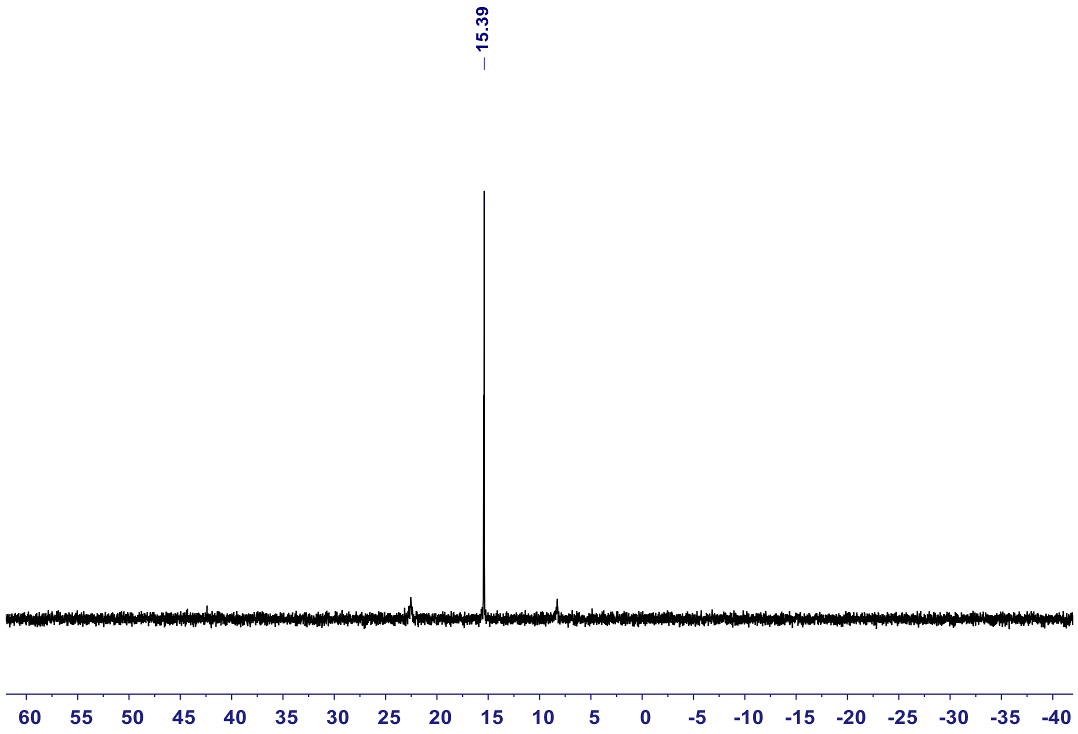

Supplementary Fig. 28 $^{31}P\{^{1}H\}$ NMR spectrum (acetone-d_6, room temperature, 161.8 MHz) of metallacycle.

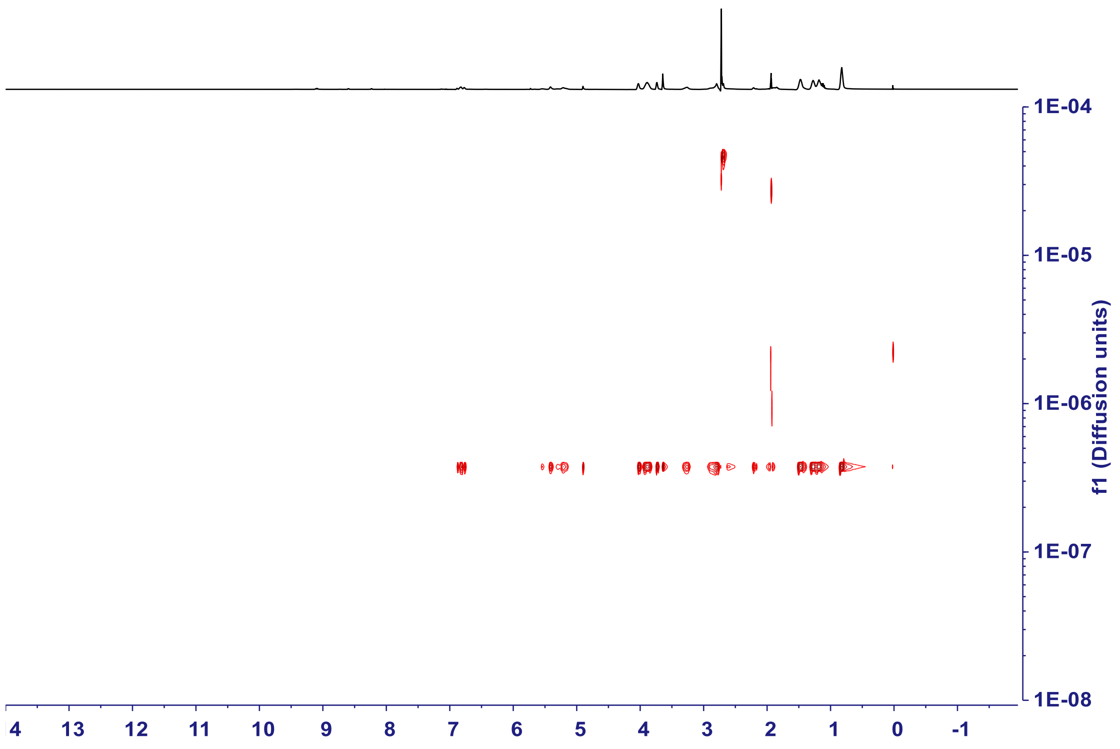

Supplementary Fig. 29 DOSY NMR spectrum (acetone-d_6, room temperature, 400 MHz) of DB24C8-based polymer.

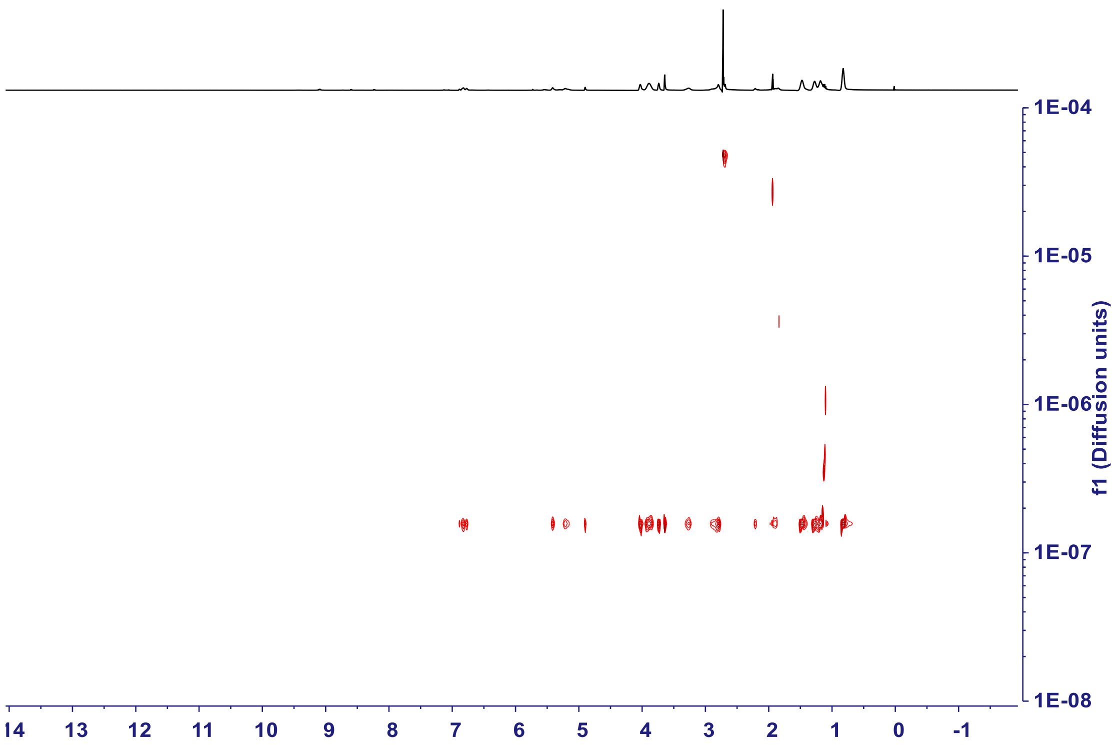

Supplementary Fig. 30 DOSY NMR spectrum (acetone-d_6, room temperature, 400 MHz) of MIN-**2**.

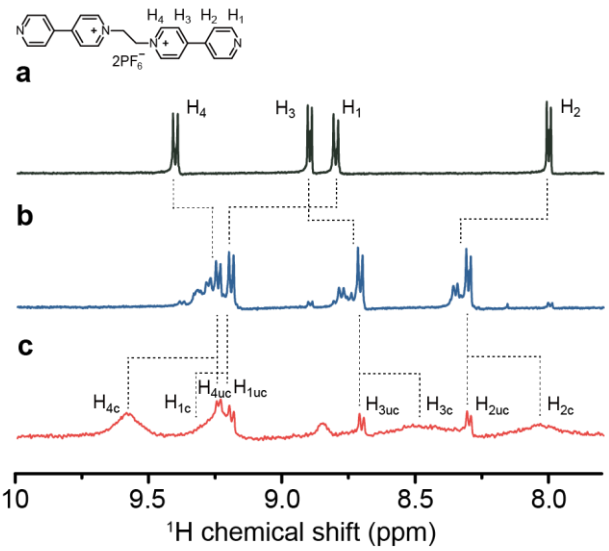

Supplementary Fig. 31 Formation of MINs. Partial ^{1}H NMR spectra (acetone-d_6, room temperature, 400 MHz) of (**a**) 1,2-bis(pyridinium)ethane, (**b**) hexagonal metallacycle, and (**c**) MIN-**3**.

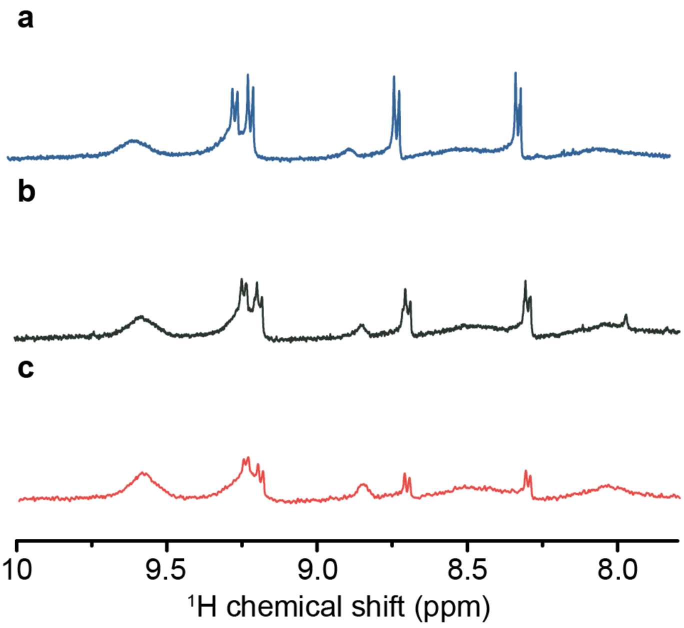

Supplementary Fig. 32 ^{1}H NMR spectra of MINs. Partial ^{1}H NMR spectra (acetone-d_6, room temperature, 400 MHz) of MIN-**1** (**a**), MIN-**2** (**b**), and MIN-**3** (**c**).

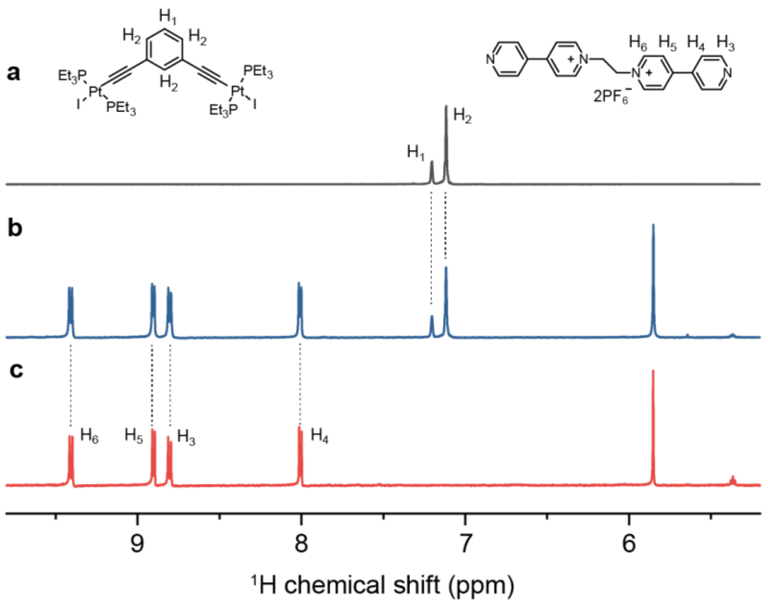

Supplementary Fig. 33 Model experiments to prove the structure of control-1. Partial ^1H NMR spectra (acetone-d_6, room temperature, 400 MHz) of the diiodo-precursor (**a**), the mixture of diiodo-precursor and bis(pyridinium) ligand (**b**), and the bis(pyridinium) ligand (**c**). After mixed the two components 2 days, all peaks belonging to the two components were well reserved in the spectrum of the mixture, and meanwhile, no peaks shift or new signals were observed. These results supported the conclusion that the two components in control-**1** were unable to form cross-linking point through metal-coordination.

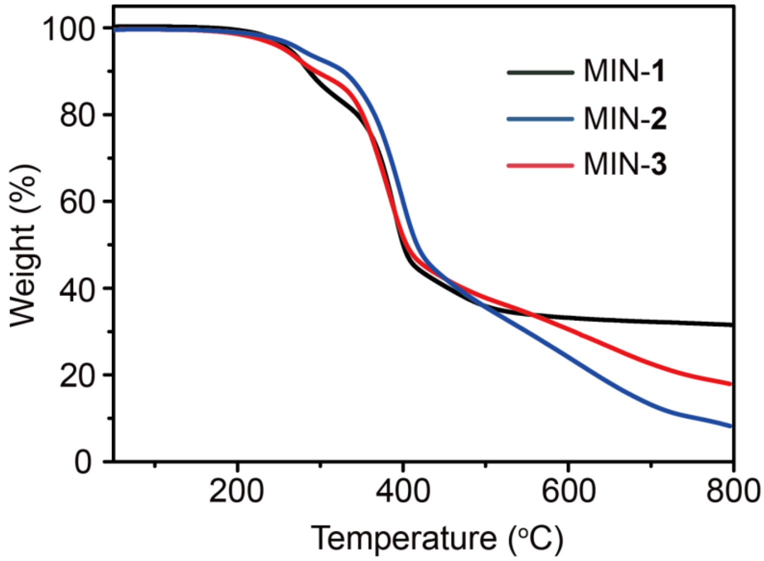

Supplementary Fig. 34 TGA curves of MINs-**1**−**3** recorded under N_2 flow (50 mL/min) with a heating rate of 20 °C/min.

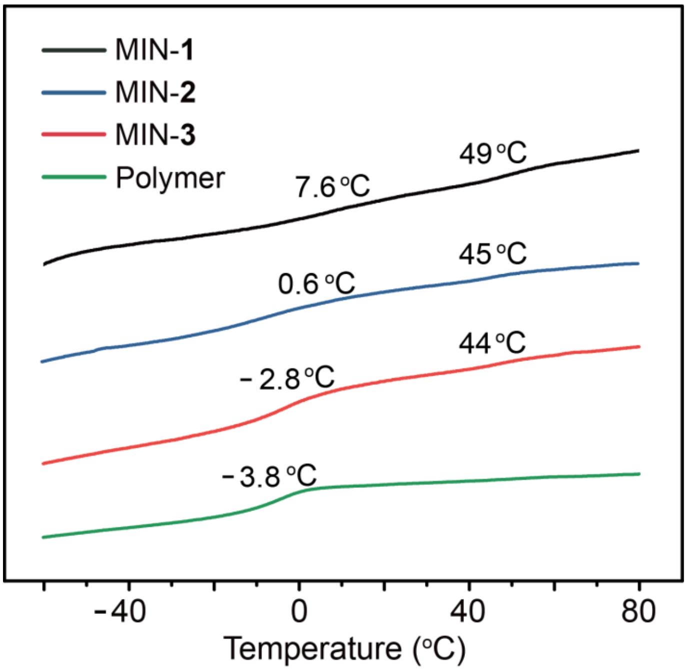

Supplementary Fig. 35 DSC curves of pure polymer and MINs-**1**–**3** scanning from −60 to 80 °C with a heating rate of 20 °C/min.

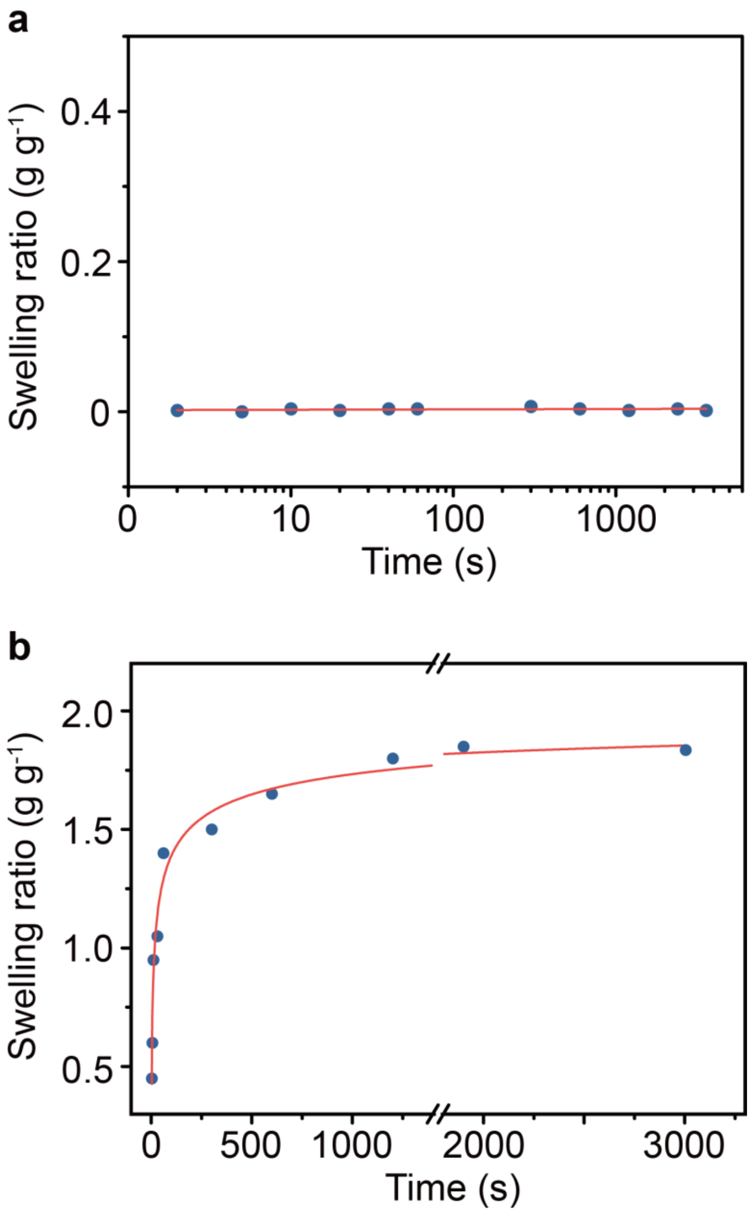

Supplementary Fig. 36 Swelling experiments. Swelling ratios of the MIN-**2** in hexane (**a**) and acetone (**b**) as a function of the swelling time.

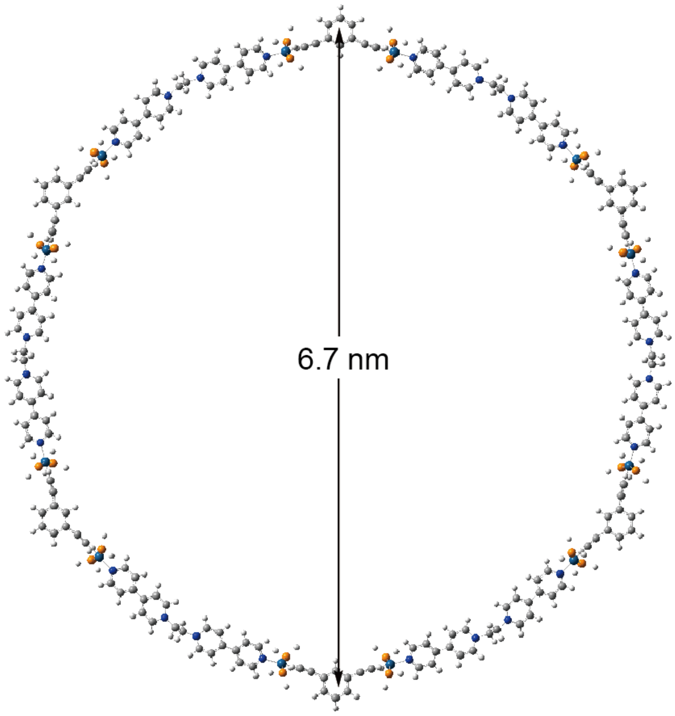

Supplementary Fig. 37 Structure of the metallacycle. The geometry structure of the metallacycle optimized by the PM6 semiempirical molecular orbital method. The PM6 calculations were carried out with Gaussian 09[12]. The geometry optimizations were performed without a solvent field in C_1 symmetry and the results are in the gas phase. To minimize computational cost, the PEt_3 ligands on platinum were modeled as PH_3 ligands.

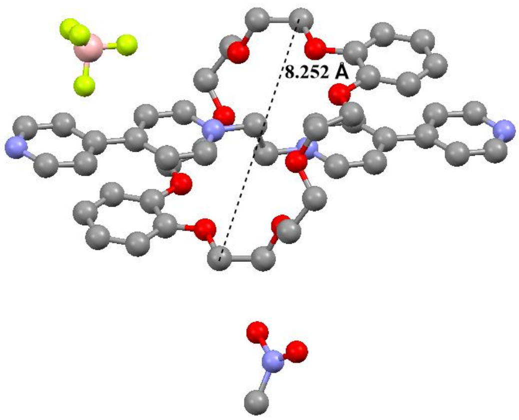

Supplementary Fig. 38 Structure of the [2]pseudorotaxane. X-Ray crystal structure of the [2]pseudorotaxane formed by DB24C8 and bis(pyridinium) ligand. The crystal data used here was reported by Loeb *et al.* in the ref. 11 with the CCDC reference number of 286500.

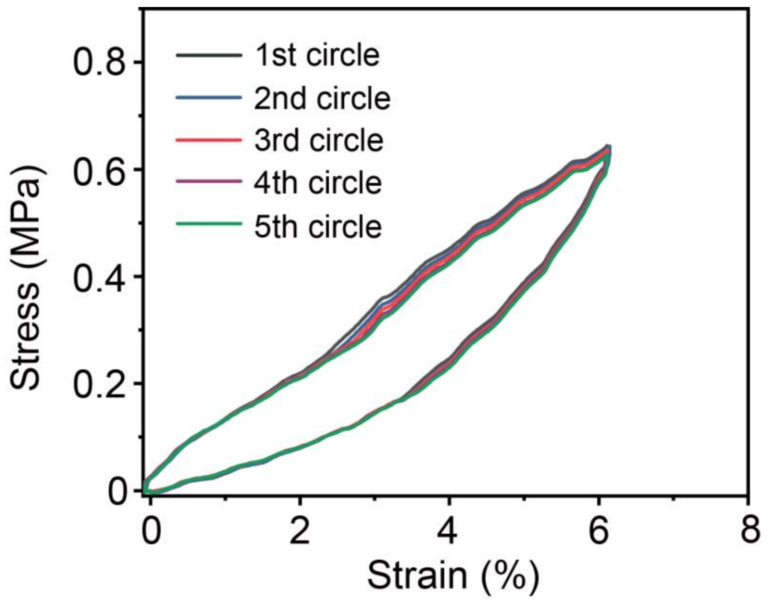

Supplementary Fig. 39 Cyclic tensile test curves of MIN-**2** at a strain of 6% under the deformation rate of 20 mm/min.

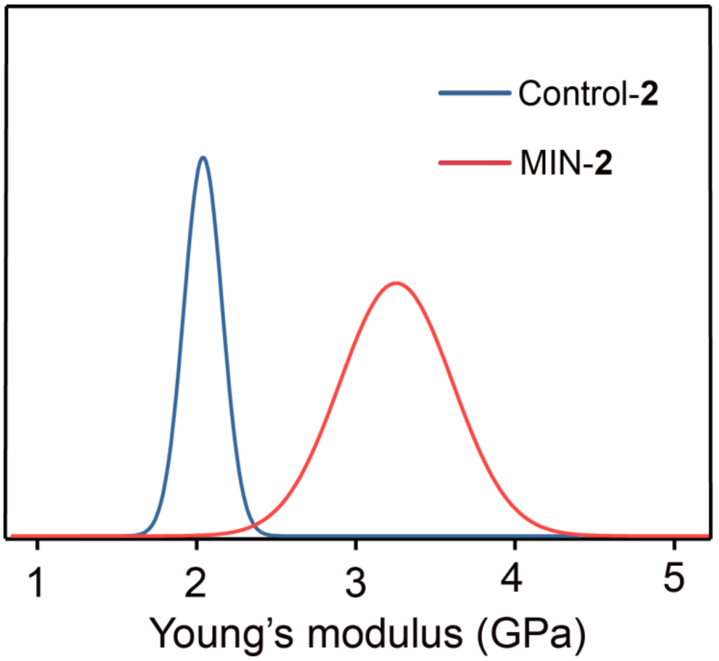

Supplementary Fig. 40 AFM modulus distribution profiles of control-**2** and MIN-**2**.

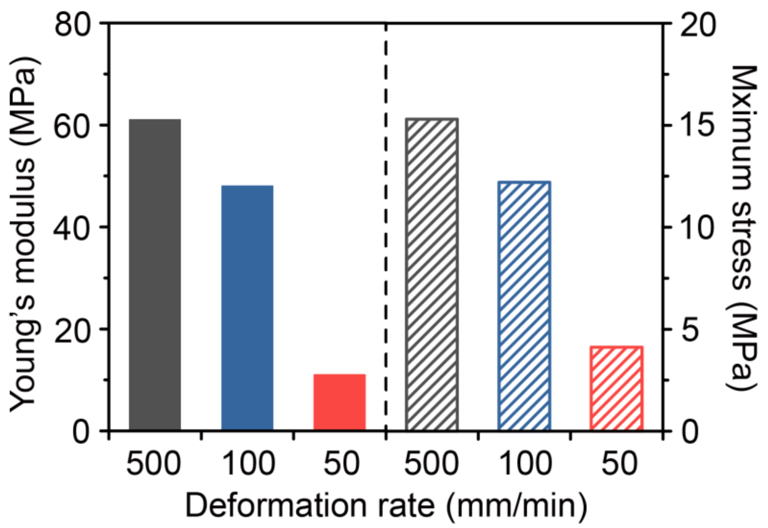

Supplementary Fig. 41 Young's moduli and maximum stress of MIN-**2** calculated from the stress–strain curves for different deformation rates.

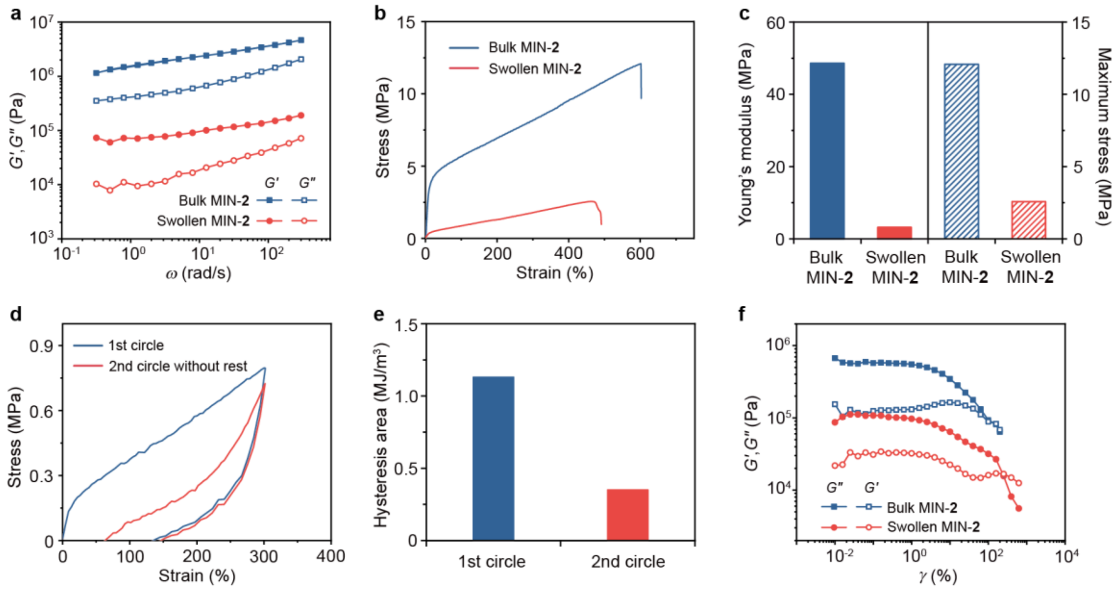

Supplementary Fig. 42 Performance comparison between bulk and swollen MIN-2. a Frequency sweep of bulk MIN-**2** (40 ˚C) and swollen MIN-**2** (25 ˚C). **b** Stress–strain curves of bulk and swollen MIN-**2** samples recorded at room temperature with a deformation rate of 100 mm/min. **c** Young's moduli and maximum stress of bulk and swollen MIN-**2** samples calculated from their stress–strain curves. **d** Cyclic tensile test curves of swollen MIN-**2** loaded at a strain of 300% recorded at room temperature with a deformation rate of 100 mm/min. **e** Hysteresis area for the two cycles of the tensile tests for swollen MIN-**2**. **f** Strain sweep curves of bulk MIN-**2** (40 ˚C) and swollen MIN-**2** (25 ˚C) at a constant angular frequency of 1.0 rad/s.

The polymeric networks in swollen state are able to weaken the inter-chain interactions such as frictional force, which is advantageous to highlight the role of molecular necklace in mechanical behaviors. For this purpose, we performed a series of tensile and rheological measurements for MIN-**2** swollen with THF (swelling ratio ~ 1.7 g/g), and compared the results with those of the bulk materials to further understand and verify the relationships between molecular necklace cross-linker and mechanical properties of MINs.

In the frequency sweep tests (Supplementary Fig. 42a), the storage modulus of the swollen sample was always higher than the loss modulus over the entire frequency range, which suggested that the network structure was well preserved in the swollen MIN-**2**, verifying the role of molecular necklace in cross-linking. Meanwhile, compared with the bulk MIN-**2**, the shear moduli of the swollen sample were much lower, which could be ascribed to the decrease in number densities of effective chains due to swelling.

Stress–strain curves (Supplementary Figs. 42b and c) provided a more sharp contrast between the bulk and swollen MIN-**2** specimens. Similar to the frequency sweep results, the Young's modulus of the swollen sample (3.22 MPa) was much lower than that of bulk material (47.3 MPa), and the breaking stress also had the same tendency. These phenomena were common for the swollen samples (Morita, H. *et al. J. Phys. Soc. Jpn.* **2009**, *78*, 041008). Besides, the breaking strain of the swollen sample (~500%) was also slightly lower than that of bulk MIN-**2** (~600%) because the polymer chains were prestretched by swelling in THF, which was also observed in the case of rubber with covalent cross-links (Morita, H. *et al. J. Phys. Soc. Jpn.* **2009**, *78*, 041008). By comparison, the breaking strain of the swollen sample in our work remained a relatively high level. The different swelling ratios should be a possible reason, but the dynamicity of molecular necklace cross-link could also contribute much to the notable stretchability.

To reveal the specific role of molecular necklace in swollen MIN-**2**, cyclic tensile tests were also conducted (Supplementary Fig. 42d and e). Similar to bulk MIN-**2**, the initial circle showed a large hysteresis area, indicative of a pronounced energy dissipation. Furthermore, the second circle without rest exhibited a significant residual strain as well as a markedly reduced hysteresis loop, which could be interpreted by the delaying recovery of the supramolecular interactions. As mentioned above, the swollen sample could reduce the effect of interactions between polymer chains. Therefore, these results are more favorable to prove that the molecular necklace could respond to applied force through its structural change to dissipate energy and thus toughen the MINs.

The response speed of molecular necklace cross-link upon force stimulus could be reflected by the strain sweep tests (Supplementary Fig. 42f). It is well-known that the linear viscoelastic region of the strain sweep curve could be used to evaluate the critical strain where the network structure starts to destroy. The destroyed structure in our work should be related to the damage of the molecular necklace cross-link. Here, we selected the tolerance range of 10% deviation for *G*′ around the plateau value as the standard to assess the strain, and the values for swollen and bulk MIN-**2** samples were measured to be 1.0% and 2.5%, respectively. The lower strain value for the swollen sample indicated a faster response of the molecular necklace cross-link. Because the chain in the swollen sample was prestretched by the solvent and thus the force could concentrate on the molecular necklace more quickly. Based on these results, we also speculate that: for the bulk MIN-**2**, the initial 1.5% (2.5% – 1.0%) shear strain is mainly attributed to the stretching of polymer chain, and after that, the force starts to concentrate on the molecular necklace cross-link.

In summary, through the property investigation of the swollen sample, the effective cross-linking role of molecular necklace was further proved. Moreover, benefiting from reducing the effect of inter-chain interactions, the dynamicity of the molecular necklace upon stress and its influence on the stretchability and toughness of the MINs were revealed more clearly. In particular, more detailed information about the shear strain responsiveness of the molecular necklace cross-link in the bulk MIN-**2** could be assessed based on the strain sweep comparison between the bulk and swollen MIN-**2** samples.

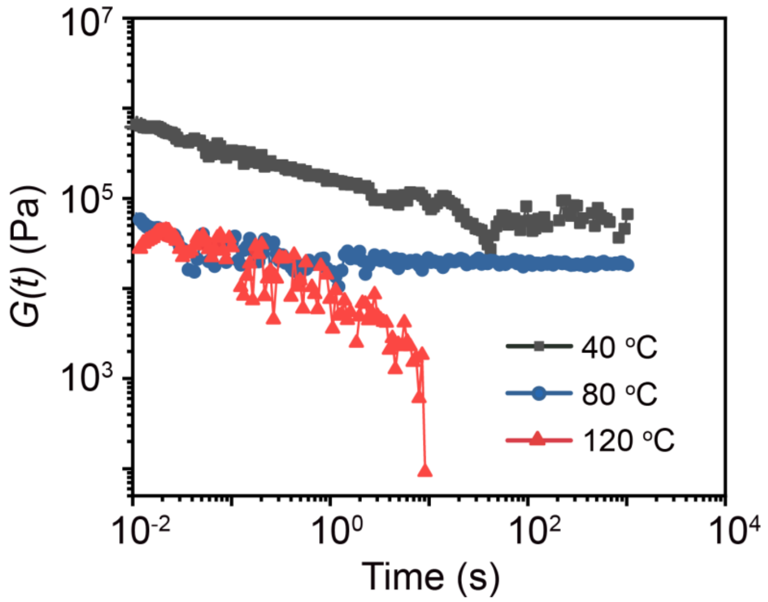

Supplementary Fig. 43 Stress relaxation curves of control-**2** at different temperatures.

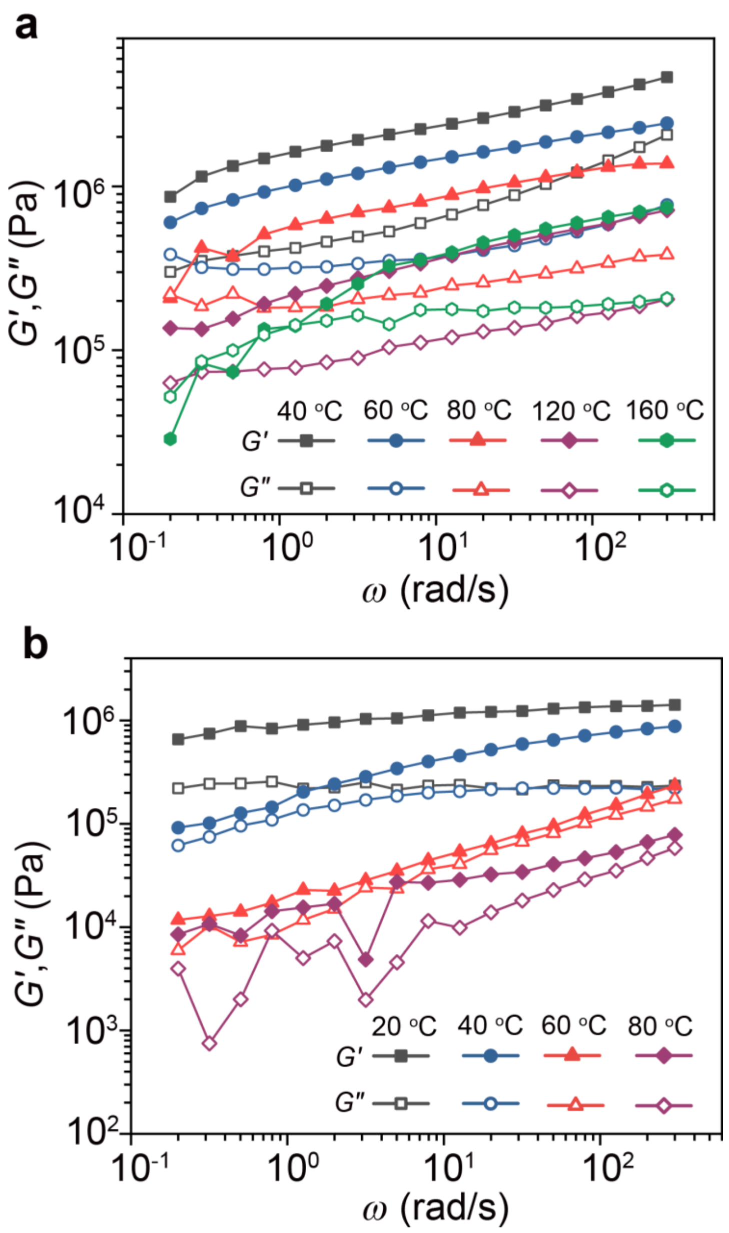

Supplementary Fig. 44 Frequency sweep of MIN-**2** (**a**) and control-**2** (**b**) at different temperatures.

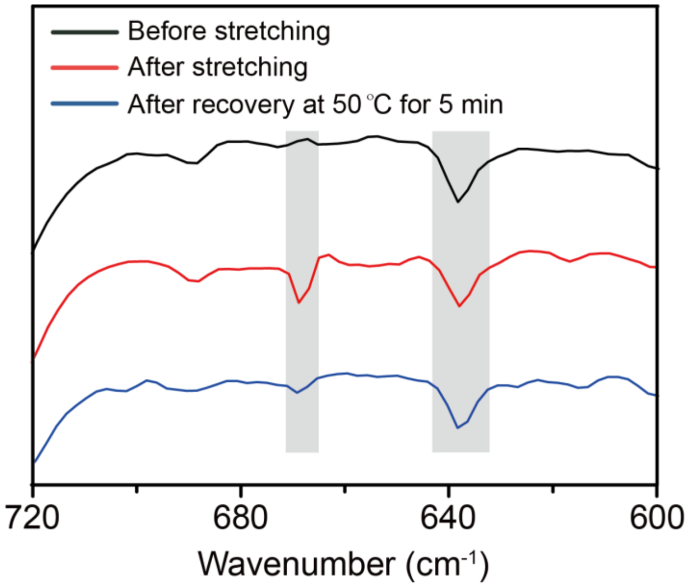

Supplementary Fig. 45 ATR-FTIR spectra. ATR-FTIR spectra of MIN-**2** to detect its structural changes upon stress. Compared with virgin sample, the spectrum of the sample after being stretched showed a peak around 669 cm^{-1}, which might be assigned as the out-of-plane C-H bending vibrations of the H_1 on bis(pyridinium) ligand without coordination. Once the ligand coordinated with platinum, corresponding peak would be shifted. Hence, the occurrence of the peak after being stretched indicated that dissociation of the coordination bond occurred upon stress. Furthermore, the dissociated coordination could basically recovered in 5 min at 50 °C.

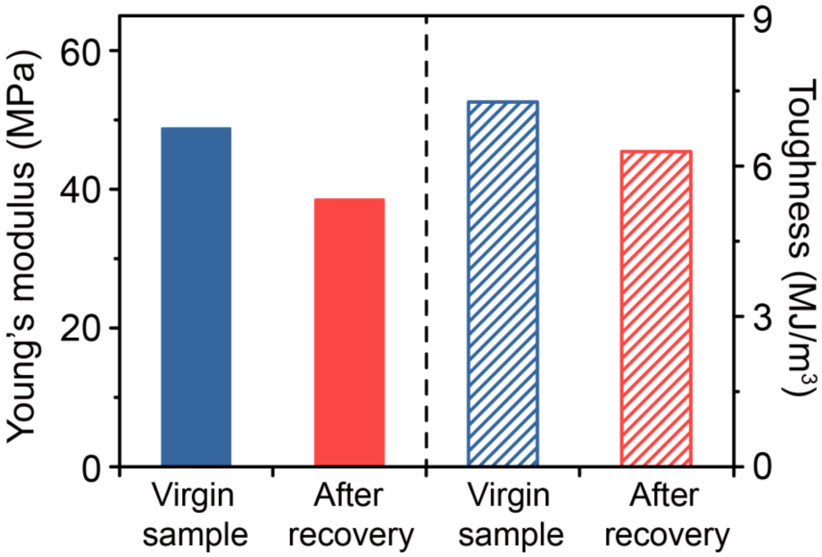

Supplementary Fig. 46 Young's moduli and toughness of MIN-**2** calculated from the results of the cyclic tensile tests in Fig. 5c.

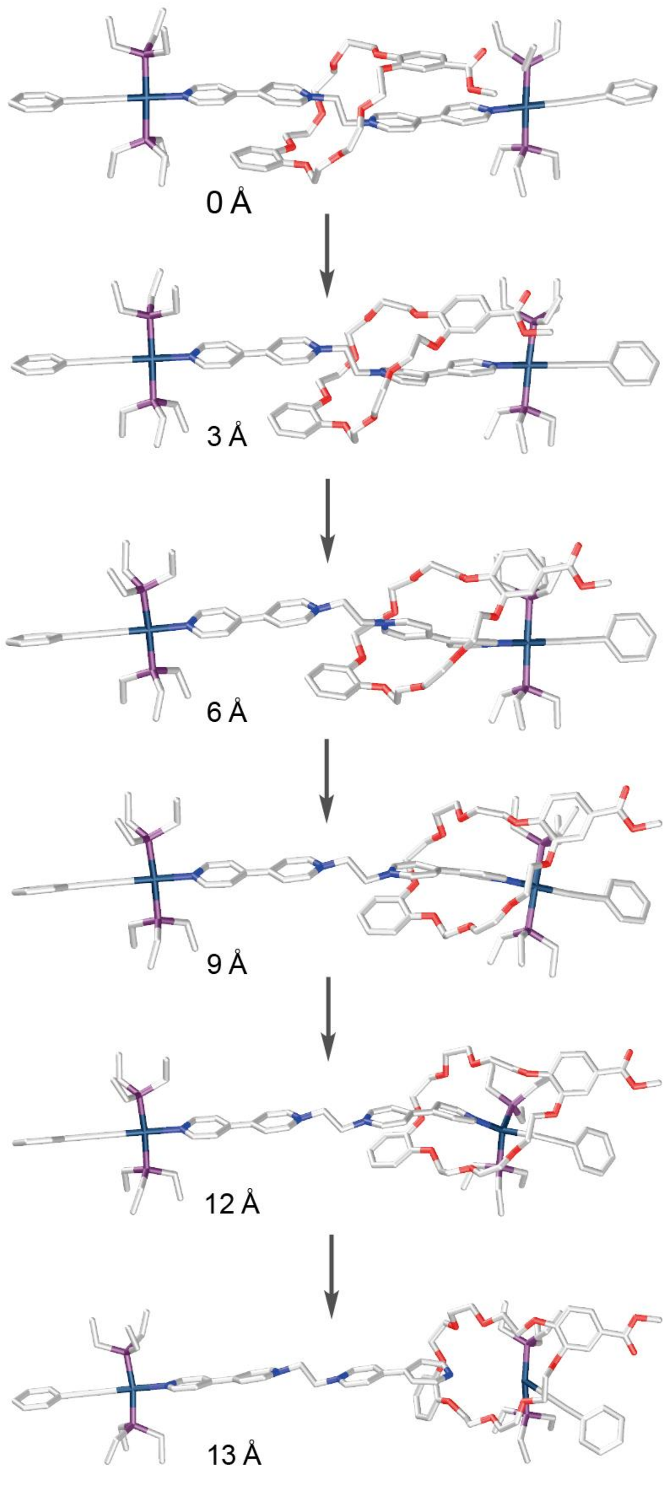

Supplementary Fig. 47 CoGEF structures of a segment of molecular necklace with different displacements.

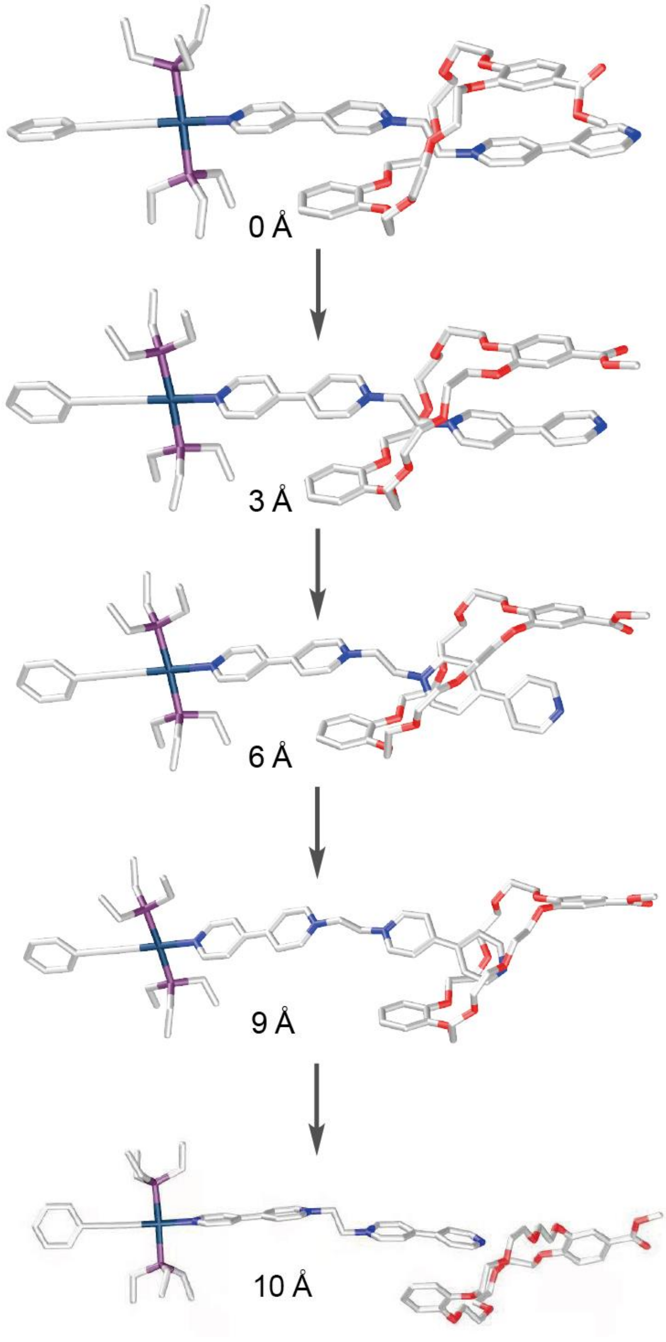

Supplementary Fig. 48 CoGEF structures of a segment of the complex in control-**2** with different displacements.

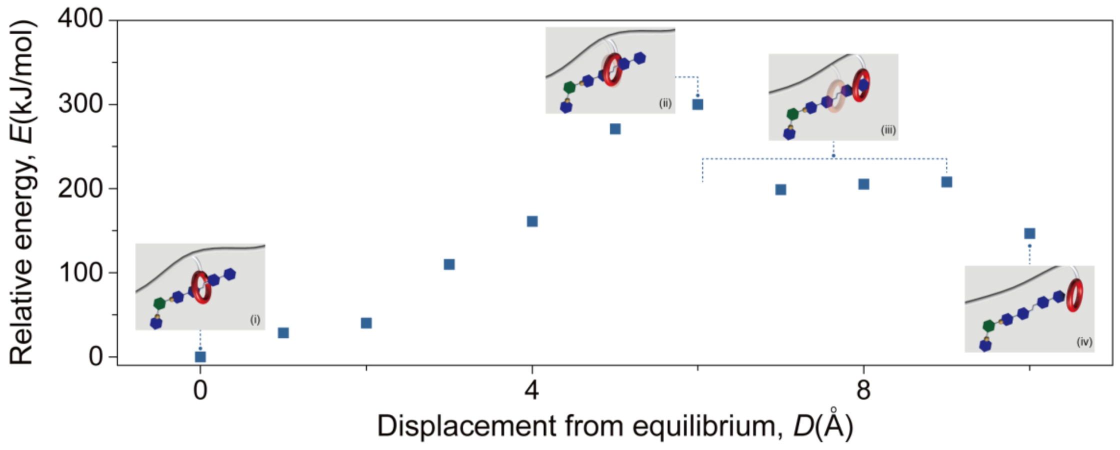

Supplementary Fig. 49 CoGEF potential as a function of stretched distance for a segment of the complex in control-**2**.

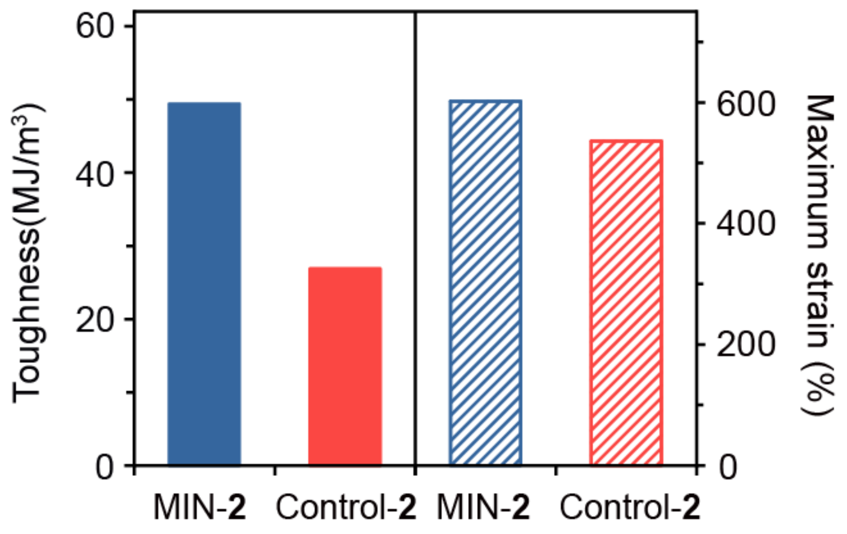

Supplementary Fig. 50 Toughness and maximum strain of MIN-**2** and control-**2** calculated from the stress–strain curves in Fig. 4e.

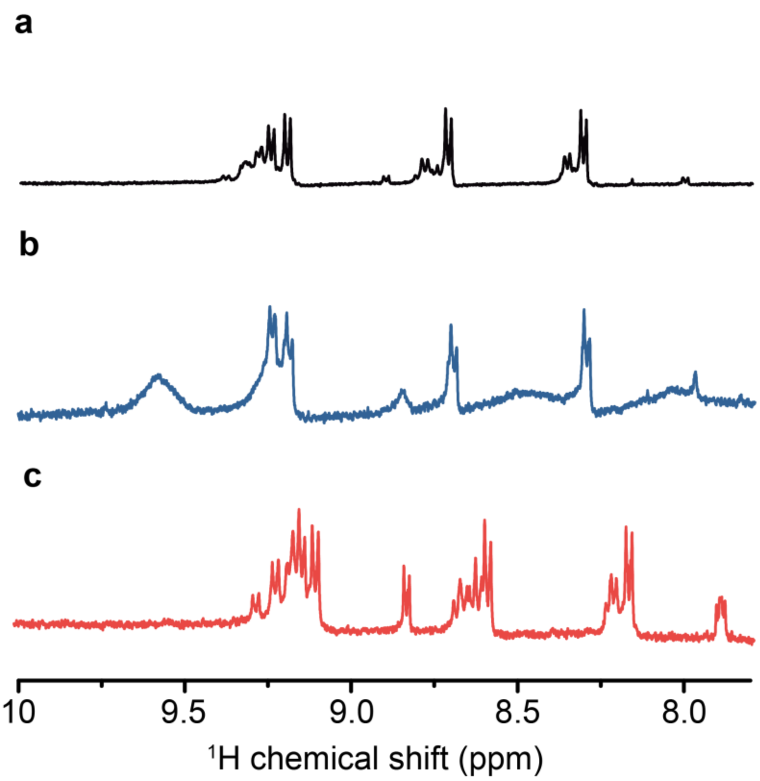

Supplementary Fig. 51 Partial ^{1}H NMR spectra (acetone-d_6, room temperature, 400 MHz) of pure metallacycle (**a**) and MIN-**2** before (**b**) and after (**c**) the treatment of KPF_6.

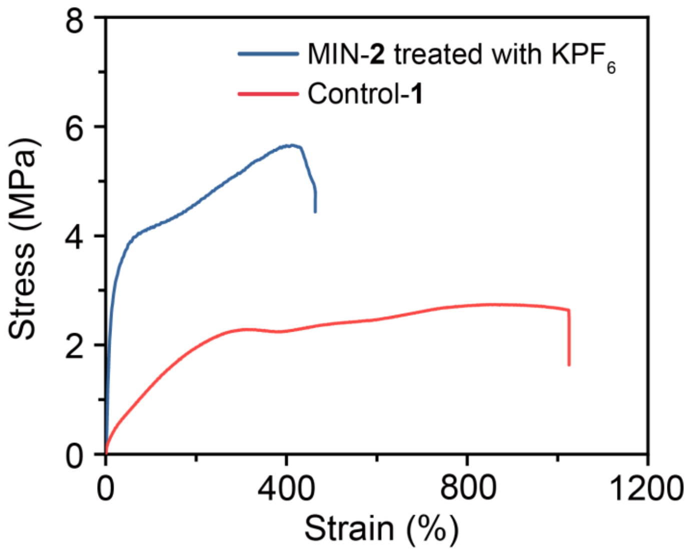

Supplementary Fig. 52 Stress–strain curves of MIN-**2** treated with KPF_6 and control recorded with a deformation rate of 100 mm/min.

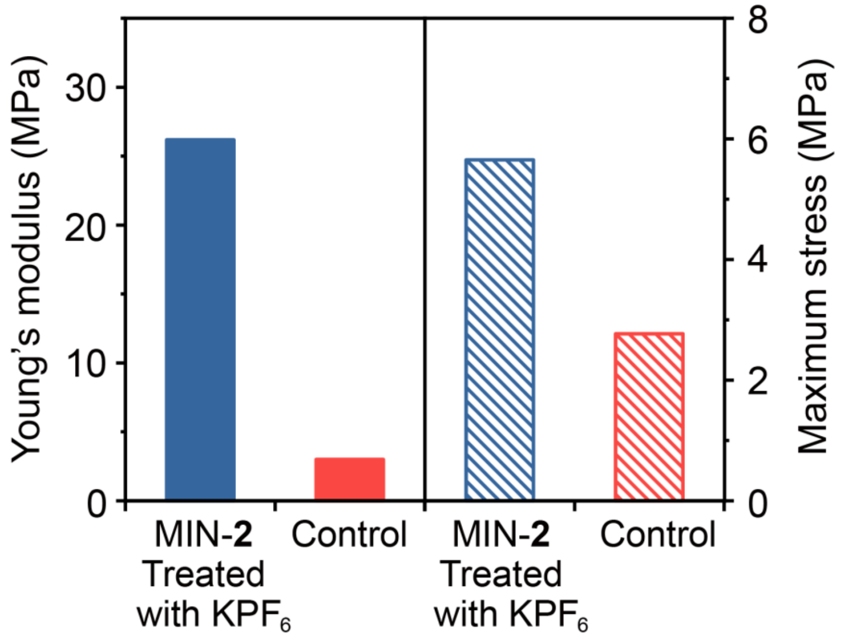

Supplementary Fig. 53 Young's moduli and maximum stress of MIN-**2** treated with KPF_6 and control.

Supplementary References

1. Chang, A. B. et al. Design, synthesis, and self-assembly of polymers with tailored graft distributions. *J. Am. Chem. Soc.* **139**, 17683−17693 (2017).

2. Wu, L., He, Y. M. & Fan, Q. H. Controlled reversible anchoring of η^6-arene/TsDPEN-ruthenium(II) complex onto magnetic nanoparticles: a new strategy for catalyst separation and recycling. *Adv. Synth. Catal.* **353**, 2915−2919 (2011).

3. Sembera, F. et al. Metal complexes with very large dipole moments: the anionic carborane nitriles 12-NC–$CB_{11}X_{11}^-$ (X = H, F, CH_3) as ligands on Pt(II) and Pd(II). *Inorg. Chem.* **55**, 3797−3806 (2016).

4. Loeb, S. J. & Wisner, J. A. A new motif for the self-assembly of [2]pseudorotaxanes; 1,2-Bis(pyridinium)ethane axles and [24]crown-8 ether wheels. *Angew. Chem. Int. Ed.* **37**, 2838–2840 (1998).

5. Yang, H. B. et al. A highly efficient approach to the self-assembly of hexagonal cavity-cored

tris[2]pseudorotaxanes from several components via multiple noncovalent interactions. *J. Am. Chem. Soc.* **129**, 14187–14189 (2007).

6. Li, S. et al. Self-assembly of triangular and hexagonal molecular necklaces. *J. Am. Chem. Soc.* **136**, 5908–5911 (2014).

7. Breßler, I., Kohlbrecher, J. & Thünemann, A. F. SASfit: a tool for small-angle scattering data analysis using a library of analytical expressions. *J. Appl. Crystallogr.* **48**, 1587–1598 (2015).

8. Beaucage, G. Approximations leading to a unified exponential/power-law approach to small-angle scattering. *J. Appl. Crystallogr.* **28**, 717–728 (1995).

9. Beaucage, G. Small-angle scattering from polymeric mass fractals of arbitrary mass-fractal dimension. *J. Appl. Crystallogr.* **29**, 134–146 (1996).

10. Beyer, M. K. The mechanical strength of a covalent bond calculated by density functional theory. *J. Chem. Phys*. **112**, 7307–7312 (2000).

11. Loeb, S. J., Tiburcio, J., Vella, S. J. & Wisner, J. A. A versatile template for the formation of [2]pseudorotaxanes. 1,2-Bis(pyridinium)ethane axles and 24-crown-8 ether wheels. *Org. Biomol. Chem.* **4**, 667–680 (2006).

12. Frisch, M. J. et al. Gaussian09 revision C.01 (Gaussian Inc., 2009).

13. Becke, A. D. Density-functional thermochemistry. III. The role of exact exchange. *J. Chem. Phys.* **98**, 5648 (1993).

14. Lee, C., Yang, W. & Parr, R. G. Development of the Colle-Salvetti correlation-energy formula into a functional of the electron density. *Phys. Rev. B: Condens. Matter Mater. Phys.* **37**, 785 (1988).

15. Petersson, G. & Al-Laham, M. A. A complete basis set model chemistry. II. Open-shell systems and the total energies of the first-row atoms. *J. Chem. Phys.* **94**, 6081–6090 (1991).

16. Hay, P. J. & Wadt, W. R. *Ab initio* effective core potentials for molecular calculations. Potentials for K to Au including the outermost core orbitals. *J. Chem. Phys.* **82**, 299 (1985).
